# Evaluating confounding in rare variant genome wide association studies

**DOI:** 10.1038/s41467-026-73776-9

**Published:** 2026-05-29

**Authors:** Aimee L. Hanson, Gareth J. Griffith, Si Fang, Neil M. Davies, George Davey Smith, Daniel J. Lawson, Gibran Hemani

**Affiliations:** 1 Medical Research Council Integrative Epidemiology Unit, Department of Population Health Sciences, Bristol Medical School, University of Bristol, Bristol, United Kingdom; 2 Division of Psychiatry and Department of Statistical Science, University College London, London, United Kingdom; 3 Department of Public Health and Nursing, Norwegian University of Science and Technology, Trondheim, Norway; 4 School of Mathematics, University of Bristol, Bristol, United Kingdom

**Keywords:** Population genetics, Genome-wide association studies, Rare variants

## Abstract

The theorised risk that confounded rare variant associations will emerge from population based genetic studies has not been investigated empirically. Here, we use 306,991 sequenced exomes from the UK Biobank to demonstrate that recent demography is poorly captured by common and rare variant principal components, and accounting for haplotype sharing does not eliminate false-positive rare variant associations with non-heritable spatially structured traits. Through re-analysis of 155 phenotypes in siblings, we show a trend of higher effect estimates bias for non-uniformly distributed traits, suggesting population stratification is most pervasive in these settings. Despite its spatial structure, bias of rare variant associations with height appeared most strongly influenced by assortative mating. We explore the risk of elevated false discovery rates for recent variants private to extended families sharing polygenic liability to extreme phenotypes, as well as through local linkage with common causal variants. Overall, we consider the complex confounding mechanisms that can impact rare variant studies and demonstrate family-based approaches can enable important sensitivity analyses.

## Abstract (web summary)

Several mechanisms can confound rare variant associations in GWAS of unrelated individuals. Here, the authors empirically examine these confounding factors and demonstrate important sensitivity analyses to assess the validity of these.

## Introduction

Rare variants detected from sequencing studies are often predicted to exhibit large and disruptive effects on biological function^1–4^, making them informative in understanding the molecular underpinnings of human physiology and disease. However, several mechanisms may confound rare variant associations from genome-wide association studies (GWAS) of unrelated individuals. Relative to common polymorphisms capturing the broad temporospatial trajectories of ancient human genealogies^5^, emerging rare variants reflect more recent changes to population structure, are more likely to be subpopulation or lineage specific, and show finer-scale geographical clustering^6–9^. Demographic effects, including population stratification, assortative mating, and indirect genetic effects, are known to bias common variant effect estimates from GWAS and derived polygenic scores (Supplementary Fig. 1), with consequences for interpretability and causal inference^10–15^. Simulation studies have provided strong theoretical grounds that rare variants are even more susceptible to confounding by fine-scale regional demographic effects, most acutely in situations where narrow allelic distributions coincide with independent but similarly spatially structured environmental, socioeconomic or technical factors influencing a trait^16–18^. In these cases, when population structure is recent and granular, adjusting for it by standard methods of fitting principal components (PCs) or genetic relationship matrices (GRM) within linear mixed models (LMM)^19–21^ appears ineffective^16,17,22,23^. Haplotype-derived ancestry components (HC) better capture fine-scale population structure^24,25^, however, whether HC fitting may reduce bias in rare variant analyses is unclear. With the increasing availability of sequenced population cohorts, empirical data now exist to investigate the extent of confounding in rare variant studies and methods to address it.

Here, we leveraged the power of 306,991 whole exome sequenced (WES) individuals from the UK Biobank (UKB) to assess the performance of common and rare variant PCs and HCs in capturing population structure and correcting for confounding by population stratification in rare variant GWAS. We assess potential bias in 1939 previously reported population-based rare variant-trait associations^26^ by re-estimating effects using a within-sibship model in 38,601 first-degree siblings, wherein the random inheritance of genetic variants from parents to offspring precludes many forms of confounding by demographic and indirect genetic effects^10,15^. We use geographical methods to assess the spatial clustering of rare variants and studied traits to assess risk of spatial confounding, and GWAS of polygenic risk scores to examine evidence of confounding due to linkage disequilibrium between rare and common causal variants, and polygenic effects acting within families. We also conduct simulations to investigate the risk of detecting strongly confounded associations with very recent rare variants carried by a single lineage, in the absence of correction for highly granular relatedness structure. Overall, we demonstrate the utility of within-sibling methods to detect biasing effects in rare variant GWAS, and present sensitivity analyses to identify unreliable single variant associations.

## Results

### Rare variant PCs capture some unique population structure

**Fig. 1:**
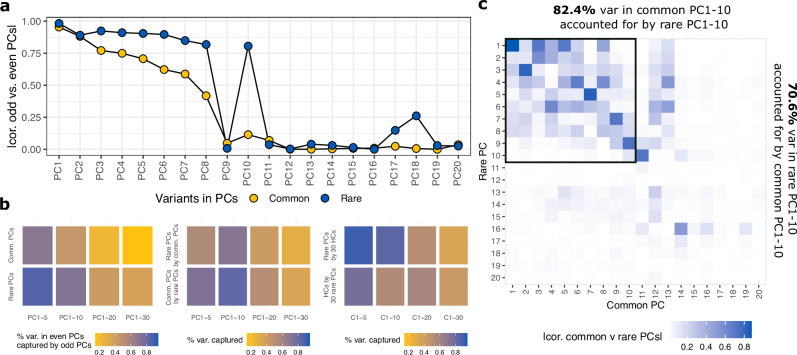
Rare variant PCs capture similar sources of genomic variation to common variant PCs, as well as chromosome specific genomic structure. **a** Absolute Pearson’s correlation between common (yellow) and rare (blue) PCs generated using odd or even autosomes separately in 306,991 UKB individuals of European ancestry. **b** Heatmaps showing percent variance in common or rare PC sets, or haplotype components, explained by combinations of other component sets. **c** Absolute Pearson’s correlation between the first 20 common and rare PCs. PC, principal component; HC, haplotype component. Source data are provided in a source data file.

Genomic PCs were developed to correct for population structure in GWAS^21^, but capture shared variation with a multitude of causes, including population variation shared between individuals, genomic variation shared between SNPs (including LD and genomic structure), and noise. The efficacy of PCs depends on the granularity of population structure and the frequency of variants included in their construction^17^, and often tails off as later PCs start to capture local genomic structure that manifests due to LD^27,28^. To compare the performance of rare (rPCs) and common variant-derived (cPCs), we split the genome into odd and even chromosomes and report on the variance odd chromosomes explain in the even, measuring the veracity of each PC set in capturing population over genomic variation^24^. Autosomal and split-chromosome PCs were generated for 306,991 UKB participants of European ancestry using two sets of variants in approximate linkage equilibrium, including: 1) 617,375 (total) rare WES variants at <1% MAF and >50 minor allele count (MAC), and 2) 147,604 (total) common variants at >1% minor allele frequency (MAF). Supplementary Fig. 2 compares cPCs to rPCs, with the latter exhibiting a cleaner clustering structure. To quantify this, we compared odd and even chromosome genomic variation, finding less genomic structure captured by rPC1-8 than cPC1-8 (Supplementary Fig. 3), the former also showing higher correlation between chromosome sets (e.g., *r* = 0.82, versus *r* = 0.42 for common PC8; Fig. 1a), implying a more consistent signal. Weaker correlations were observed for later PCs across both variant sets as chromosome-specific structure began to contribute disproportionately to explained variance (Fig. 1a, Supplementary Fig. 3). As PC order may differ across sets, we calculated the percent of the variance captured by each PC set accounted for by the others through canonical variates. Odd PC1-10 explained 72.4% and 45.3% of the variance captured by even PC1-10 for rPCs and cPCs, respectively (Fig.1b), suggesting that early rPCs more robustly capture population structure. Autosomal rPC1-10 captured 82.4% of the variance in cPC1-10, whereas cPC1-10 explained 70.6% of the variance in rPC1-10 (Fig.1b, c). rPCs performance dropped substantially if variants were not first LD pruned (only 22.7% of the variance in unpruned odd rPC1-10 was explained by unpruned even rPC1-10).

A recent assessment of HCs, generated using chromosomal regions of inferred ancestral origin, suggested these capture more recent demography than variant-based PCs^24^. HC1-30 generated for UKB participants captured >84% of the variance in rPC1-10 (Fig.1b) and could strongly predict these using linear regression (*R*^*2*^ = 0.56-0.97; Supplementary Fig. 4). Conversely, rPC1-30 captured 54% of the variance in HC1-10 (Fig. 1b), and poorly predicted HC1,6,7,8 and 9 (*R*^*2*^ < 0.35; Supplementary Fig. 4). Overall, although rPCs generated using variants of the applied frequency range appeared to capture some unique background genomic variation over cPCs, and were less susceptible to absorbing local genomic structure, the strong correlation between rPCs and cPCs suggests that rPCs predominantly also inform on the ancient genomic variation cPCs absorb and not the recent population structure captured by HCs.

### Rare and common variant PCs insufficiently control population stratification in rare variant GWAS

**Fig. 2:**
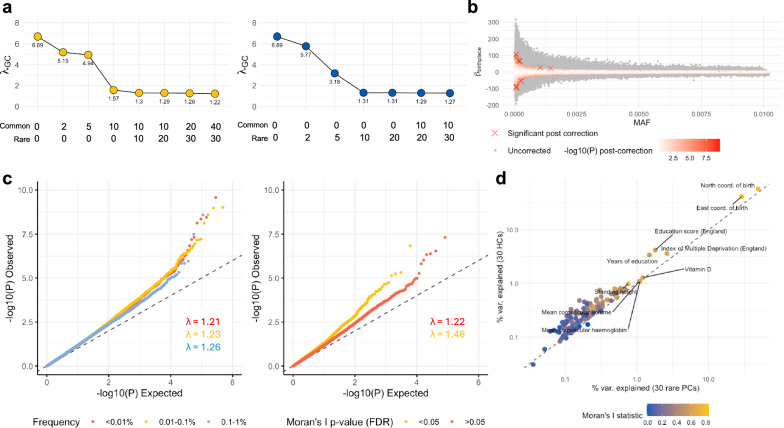
Population structure inflates rare variant effect estimates in negative control GWAS of birthplace with fitting of common or rare variant derived PCs. **a** Genomic inflation factor (*λ*_GC_) from the rare variant GWAS of the northing coordinate of birthplace with successive inclusion of common or rare variant derived PCs. **b** Per-variant SNP effect estimates from the rare variant GWAS of northing coordinate on birthplace pre and post correction for a maximal set of 40 common and 30 rare PCs, plotted against variant minor allele frequency (MAF) and coloured by –log10(P-value) as taken from the corrected GWAS. Rare variants that remain significantly associated with birthplace at a genome-wide significant threshold of *P* < 5 × 10^−8^ are indicated by crosses. **c** Q-Q plots showing observed vs expected –log_10_(P-value) from GWAS with correction for 40 common and 30 rare PCs for variants split by MAF (left; red, MAF < 0.01%; yellow, MAF 0.01-0.1%; blue, MAF 0.1-1%) or evidence for spatial autocorrelation (right; yellow, Moran’s I test P_FDR_ < 0.05; red, P_FDR_ > 0.05; only chromosome 1 variants shown). **d** Comparison of the variance explained in 155 quantitative traits by 30 rare PCs, or 30 HCs fitted in a linear regression, with traits coloured according to Moran’s I statistic. Source data are provided in a source data file. PC, principal component; HC, haplotype component. Figure 2d.

To compare the utility of rPCs and cPCs in controlling for genomic inflation due to population stratification in rare variant GWAS, we tested for rare variant associations with a negative control trait, the north co-ordinate of birthplace, in 279,390 UK-born individuals of European ancestry. Without PC inclusion, rare variants genome-wide showed strong birthplace associations due to the non-uniform geographical distribution of minor alleles, resulting in substantial genomic inflation (λ = 6.69, Fig. 2a, Supplementary Fig. 5a, d). rPC1-10 performed better than cPC1-10 in reducing genomic inflation (to λ = 1.31 and λ = 1.57, respectively), with no substantial improvement seen upon adjusting for both sets combined (λ = 1.30; Fig. 2a). Variable inclusion of later PCs (up to a maximum of 40 cPCs and 30 rPCs) failed to completely control genomic inflation (λ = 1.22; Fig. 2a), and genome-wide significant associations with birthplace remained for very rare variants exhibiting spatial restriction (0.008-0.1% MAF; Fig. 2b, Supplementary Fig. 5b, e). Genomic inflation following maximal PC correction was comparable for rare variants grouped by frequency (<0.01%, 0.01–0.1%, 0.1–1%) but more pronounced for those showing spatial clustering based on Moran’s I statistic, a measure of spatial autocorrelation (λ = 1.46 for Moran’s I P_FDR_≤0.05, λ = 1.22 for P_FDR_ > 0.05; assessed using chromosome 1 variants only; Fig. 2c). Inclusion of the HC1-30 reduced genomic inflation to ~1 (λ = 1.01) though strong associations remained with single, highly structured rare variants (Supplementary Fig. 5c, f).

The non-uniform distribution of GWASed traits may similarly result in confounded rare variant associations when recent demography is insufficiently controlled. When correlating 163 quantitative traits in the UKB with the north co-ordinate of birthplace, strong correlations were seen with the index of multiple deprivation and pack-years smoking, and negative correlations with standing height and years in education (Supplementary Fig. 6). We compared the percentage of variance in measured traits explained by rPC1-30 and HC1-30 using linear modelling. The variance explained was minimal, but highest for those traits showing substantial spatial autocorrelation by Moran’s I statistic, including education score, deprivation index, vitamin D, mean corpuscular volume/haemoglobin, and standing height. HCs performed better than rPCs in capturing the variance due to demography for these traits, and explained an additional 8.6% of the variance in birthplace (a total of 57.7%; Fig. 2d). Overall, no combination of the generated cPCs and rPCs was sufficient to completely control genomic inflation in rare variant GWAS of a non-heritable spatially stratified trait, suggesting a potential for demographic confounding by residual, fine-grained population structure. Rare variants showing spatial clustering were particularly susceptible to confounding, and fitting neither HCs nor PCs could eliminate false-positive associations with these.

### Re-estimation of rare variant associations within sibships indicates bias

Given that PCs failed to completely control demographic confounding in a negative control rare variant GWAS, we tested for bias in published rare variant associations. We applied a described within-sibship (WS) model^15^ to 38,601 UKB full siblings of European ancestry to re-estimate rare putative loss of function (pLOF) variant effects on quantitative traits reported by Backman et al.^26^ in their exome sequencing analysis of 454,787 UKB participants^26^. WS models are substantially less biased by demographic and indirect genetic effects, which are held more constant when modelling the transmission of alleles within a family than across members of a demographically varying population^10,28,29^. We tested 702 rare (MAF 0.009–1% with >20 MAC across individual siblings) pLOF and deleterious missense variants with modest to strong (*P* < 1 × 10^−5^) associations across 155 quantitative traits (a total of 1939 rare variant-trait pairs with an average (range) of *n* = 26,679 (2123–37,057) siblings per trait), comparing WS to reported population effect estimates. For consistency with the population model used, we included age, sex, age^2^, age × sex, cPC1-10 and rPC1-20 as covariates in the WS model. As anticipated, effect estimates for 42 birthplace-associated rare variants (*P* < 1 × 10^−5^ in the population GWAS above) showed strong attenuation toward the null in the WS model. The slope estimate from the linear regression of WS on population effect estimates was consistent with zero (β = 0.03, SE = 0.09), and substantially different from 1 (where slope(β) = 1 indicates perfect concordance between model estimates, *P*_ß=1_ = 4.8 × 10^−13^; Supplementary Fig. 7).

**Fig. 3:**
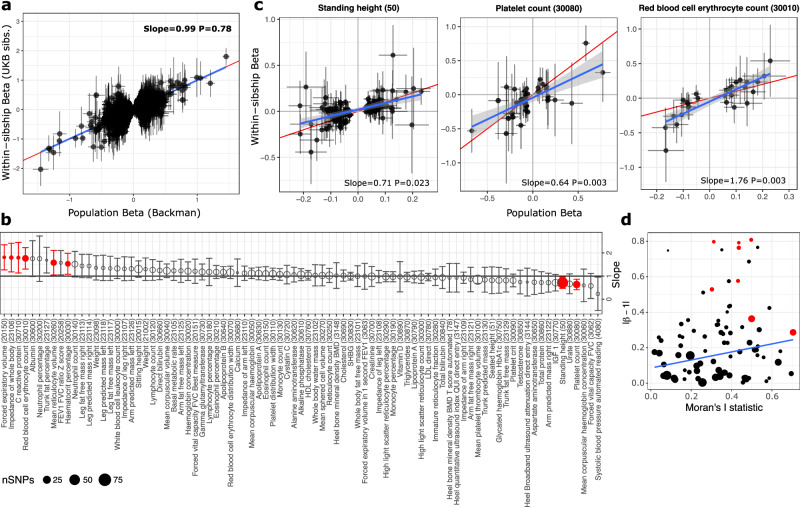
Differences in population and within-sibship (WS) derived effect estimates are seen for some trait associated rare variants. **a** Comparison between WS and population-derived rare variant effect estimates from Backman et al.^26^ for *n* = 1939 quantitative trait-associated rare variants (at *P* < 1 × 10^−5^). *P*-value of the t-statistic (*P* = 0.78) indicates the slope of the linear regression of WS on population betas across all tested variants does not differ from 1 (model concordance; red line). Error bars indicate 95% confidence intervals about each model SNP effect estimate. **b** Slopes returned for all tested quantitative traits with 8 or more associated rare variants at *P* < 1 × 10^−5^ in Backman et al.^26^ Traits with P_*β *= 1_ < 0.05 are shown in red (Forced exp. vol., *P* = 0.02; Impedance, *P* = 0.03; C-reactive protein, *P* = 0.04; RBC eryth. count, *P* = 0.003; Mean retic. vol., *P* = 0.04; Haem. percentage *P* = 0.03; Standing height, *P* = 0.02; Plat. count, *P* = 0.003), points are scaled by number of included variants (*n* = 8–90), error bars indicate 95% confidence interval about the slope estimate from the linear regression of WS on population estimates, UKB field codes are shown after trait names. **c** Example population versus WS variant effect estimate comparison and estimated linear regression slope for variants associated with standing height (*n* = 90), platelet count (*n* = 36) and red blood cell erythrocyte count (*n* = 28). Error bars indicate 95% confidence intervals about each model estimate, *P*-value of the t-statistic indicates that the regression slope differs from 1 (*P* = 0.023, *p* = 0.003, *P* = 0.003, respectively). **d** Regression of the per-trait change in slope against the trait Moran’s I statistic, traits coloured red in b are indicated. Points are scaled by the inverse squared standard error of the slope estimate. Source data are provided in a source data file and within Supplementary Data 1.

There was a high concordance between the population model and WS model effect estimates for the 1939 rare variant-trait pairs tested (ß = 0.99, SE = 0.02, *P*_ß = 1_ = 0.78; Fig. 3a, Supplementary Data 1). 135 rare variant-trait estimates showed nominal heterogeneity between models when tested independently (Cochran’s Q statistic; P_Q_ < 0.05), but did not survive control for false discovery rate (FDR) across traits. As the extent of demographic confounding, and thus estimated shrinkage, may differ across traits based on their spatial non-uniformity, we tested for shrinkage in population effect estimates on a trait-by-trait basis for the 85 traits that had > = 8 associated rare variants (Fig. 3b). Both standing height and platelet count showed evidence of substantial shrinkage in variant effects in the WS model (height ß_shrinkage_ = 0.71, SE = 0.12, *P*_ß = 1_ = 0.02; platelet count ß = 0.64, SE = 0.12, *P*_ß = 1_ = 0.003) with single variants showing effect heterogeneity (though these did not withstand FDR control; Fig. 3b, c). Conversely, effect estimates for several red blood cell parameters (haematocrit percentage, mean reticulocyte volume and red blood cell reticulocyte count), C-reactive protein, whole body impedance and forced expiratory volume were deflated compared to WS estimates, suggesting a degree of negative confounding (occurring when biasing effects act in opposition to direct genetic effects, deflating population estimates; Fig. 3b, c). Results were similar when the change in slope was re-estimated using only the top associated variant per gene, with shrinkage additionally observed for systolic blood pressure (ß = 0, SE = 0.30, *P*_ß = 1_ = 0.01), and increased across height variants (ß = 0.62, SE = 0.12, *P*_ß = 1_ = 0.003; Supplementary Fig. 8). We ran an inverse variance weighted regression to examine whether more spatially clustered traits showed greater differences between model estimates. The absolute change in slope for each within-trait model comparison (|ß−1|) was positively associated with the degree of trait spatial auto-correlation (Moran’s I statistic; ß = 0.23, SE = 0.1, *P* = 0.02, Fig. 3d). Overall, concordance in rare variant effect estimates between population and WS GWAS models suggests minimal detectable bias on a per-variant level. However, when aggregating estimates across multiple variants per trait, there was evidence that confounding factors may systematically bias population-derived rare variant effects, particularly for traits nonuniformly spatially distributed in the population.

### Bias in height effect estimates is not reduced by direct spatial measures

**Fig. 4:**
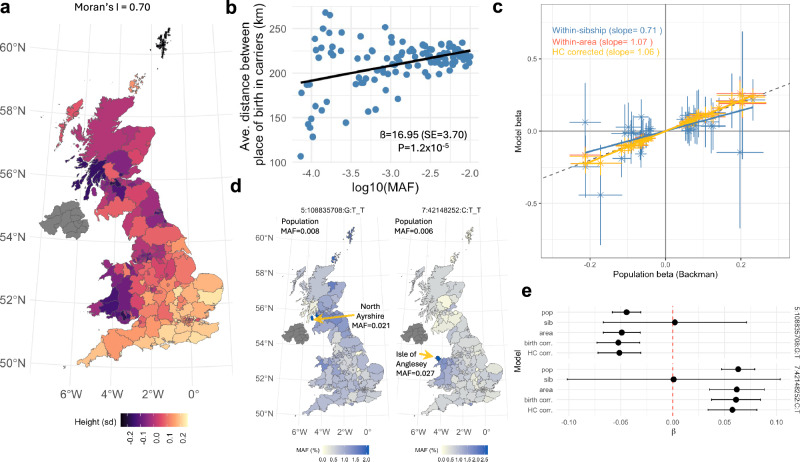
Spatial confounding does not explain bias in rare variant height effect estimates. **a** Area-specific deviation in height across European UKB participants grouped by county or unitary authority of birthplace. **b** Association between log_10_(MAF) and average Euclidean distance between the birthplace of variant carriers (slope and P-value from linear regression shown) for *n* = 116 height-associated rare variants from Backman et al.^26^ (MAF < 1%, *P* < 5 × 10^−8^). **c** Re-estimated height effects for 42 height-associated rare variants (MAC > 20) using within-sibship (blue), within-area (red) and HC corrected (yellow) models. Betas are plotted against the population model estimates from Backman et al.^26^, error bars indicate 95% confidence intervals, dashed line represents a slope of 1. **d** Area specific minor allele frequencies for two rare height associated variants showing the greatest spatial autocorrelation by Moran’s I statistic, MAFs are shown for areas of birth with the highest proportion of minor allele carriers (5:108835708:G:T T-allele carriers in North Ayrshire, *n* = 12 het., *n* = 1 hom. of *n* = 332; 7:42148252:C:T T-allele carriers, *n* = 5 het. of *n* = 93). **e** Height effect estimates taken from population (pop), within-sibship (sib), within-area (area), northing and easting corrected (birth corr.) and 150 HC corrected (HC corr.) models for the two variants in (**d**). Error bars indicate 95% confidence intervals. UK maps were created using the spdep package in R v4.4 with shapefiles taken from the December 2023 ultra-generalised UK vector boundaries from the Office for National Statistics (https://geoportal.statistics.gov.uk/datasets/countries-december-2023-boundaries-uk-buc/about). Sourced data are provided in a source data file.

In simulation studies, rare variants showing strong spatial restriction are most liable to bias due to spatial confounding when the tested trait also shows spatial patterning^16,17^. Standing height, which shows substantial spatial auto-correlation in the UKB (Moran’s I = 0.70, *P*_FDR_ = 3.2 × 10^−46^; Fig. 4a), exhibited the most rare variant associations with which to test this. To assess the spatial distribution of height-associated rare variants from Backman et al.^26^, we determined the average minor allele frequency across each of 218 counties and unitary authorities (CTYUA) across England, Scotland and Wales (see “methods” for details), assigning individuals to boundaries based on their birthplace coordinates. We derived Moran’s I statistic of spatial autocorrelation for each variant using CTYUA groupings, and the average pairwise Euclidean distance between the birthplace coordinates of carriers using *n* = 279,390, unrelated UK-born individuals of European ancestry. There was a strong positive association between variant MAF and the average distance between the carrier birthplace (in km, ß = 16.95, SE = 3.70, *P* = 1.2 × 10^−5^), but no association between MAF and Moran’s I (ß = −0.02, SE = 0.01, *P* = 0.3), suggesting CTYUA boundaries may be too coarse to capture variant clustering. Variants that exhibited the smallest average distance between birthplace in carriers ( < 150 km) were typically the most rare (MAF < 0.01%), but this was not true of all ultra-rare variants, some of which were widely geographically dispersed (Fig. 4b).

To determine whether the shrinkage observed in height effect estimates derived in siblings could be accounted for using measured spatial information, we re-estimated the direct effect of 42 rare height-associated variants (Backman et al.^26^ *P* < 5 × 10^−8^ and MAC > 20 in siblings, min MAF = 0.04%) using two spatially informed models; 1) a linear model with cluster-robust standard errors, testing whether having a minor allele dosage higher than the birthplace CTYUA average associated with height, correcting for area mean genotype; and 2) a standard population GWAS with additional inclusion of northing and easting coordinates of birth as linear covariates (see “methods” for model details). We also re-estimated population-level effects, substituting PCs with 150 HCs, which better capture fine-grained demography. To assess the extent to which covariates captured the spatial non-uniformity in height, we derived Moran’s I statistic for the residuals returned when regressing cPC1-10 and rPC1-20, HC1-150 or northing and easting coordinates of birth on measured height. All model residuals continued to show spatial patterning, though to a lesser extent than unadjusted height (PCs Moran’s I = 0.44, *P* = 3.5 × 10^−20^; HCs Moran’s I = 0.28, *P* = 2.5 × 10^−9^, N/E Moran’s I = 0.39, *P* = 2.4 × 10^−16^, Supplementary Fig. 9). Variant height effect estimates from the within-area and HC adjusted models were slightly inflated over population estimates from Backman et al.^26^ (within-area model: ß = 1.07, SE = 0.03, *P*_ß = 1_ = 0.02; HC-adjusted model: ß = 1.06, SE = 0.02, *P*_ß = 1_ = 0.01), whereas WS estimates showed shrinkage, as previously (ß = 0.71, SE = 0.13, *P*_ß = 1_ = 0.04; Fig. 4c). Substantial heterogeneity was not detected in variant effect estimates across the population, area informed or HC corrected models for any height associated variant tested, including variants showing the greatest spatial autocorrelation (5:108835708:G:T, MAF = 0.008, Moran’s I = 0.33, *P* = 1.2 × 10^−12^; 7:42148252:C:T, MAF = 0.006, Moran’s I = 0.30, *P* = 4.2 × 10^−11^; Fig. 4d, e). Thus, although rare variants associated with height exhibit substantial spatial clustering, application of HCs or area-informed models was unable to recapitulate the shrinkage observed in WS estimates. The geographical distribution of height, which is likely sufficiently captured by all area correction methods, may not be sufficiently sharp to confound rare variants associations, particularly those we had the power to assess in siblings (with >0.01% MAF). Shrinkage in WS effect estimates for rare height variants may instead indicate the systematic inflation of population-derived test statistics through assortative mating or indirect genetic effects. Supporting this, the estimated shrinkage in WS height effects expected under assortative mating (E(S_PS_) = 0.77) approximated that observed (ß = 0.71; see Supplementary Note 1). Assessment of traits exhibiting more extreme spatial restriction will be needed to more robustly assess the circumstances under which spatial confounding may arise in rare variant GWAS.

### Familial polygenic confounding may result in false-positive rare-variant associations

We considered two additional mechanisms of confounding in rare variant GWAS: 1) confounding due to local LD with causal common variants and 2) confounding due to recent relatedness. The latter, which we refer to here as familial polygenic confounding, can be considered an extreme version of confounding by cryptic relatedness (CR)^30^, and may occur in situations where de novo variants, private to a family lineage, emerge within a genomic landscape containing a high common variant burden for a polygenic trait, underlying an extreme phenotype. Such variants would track with genome-wide trait-modifying alleles and may appear associated with the measured trait at a population level until repeated recombination events over multiple generations decay the shared polygenic component between extended relatives carrying the rare variant. Both mechanisms may induce false positive associations with rare variants, which would also be expected to associate with the common variant polygenic component of the trait, presenting a means by which to detect them.

**Fig. 5:**
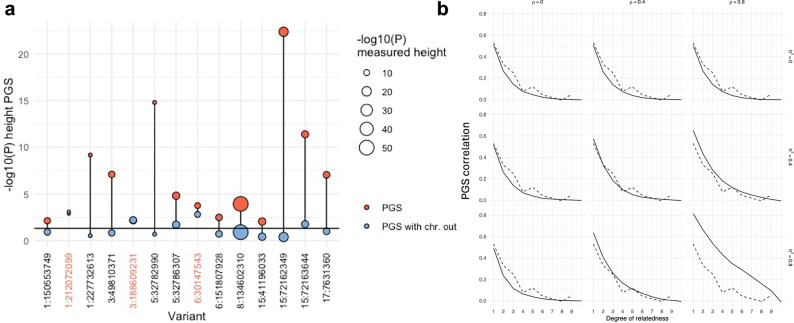
Familial polygenic confounding has the potential to bias rare variant associations. **a** Association of 14 height-associated rare variants with the common variant-derived height PGS with (red circles) and without (blue circles) inclusion of the variant chromosome. *P*-values are derived from linear regression with correction for 10 common and 20 rare PCs. Horizontal line corresponds to *P* = 0.05; variants retaining association at P_FDR_ < 0.05 are coloured red on the x-axis. Points are scaled to the *P*-value of the variant height association. **b** Correlation between the common variant-derived polygenic score for height in individuals related to differing degrees, taken from UKB participants (dashed line) or a forward-in-time simulated cohort of individuals using *n* = 500 causal common variants (solid line) under varying trait heritability (h^2^) and assortative mating (*ρ*). Sourced data are provided in a source data file.

To assess these two mechanisms in the context of height, a highly heritable polygenic trait with a large fraction explained by known GWAS loci, we derived a height polygenic score (PGS) using common variant weights from an independent cohort (capturing 30.2% and 30% of the variance in height in UKB males and females, respectively; see “methods”). We then tested 67 rare variants with standing height associations (Backman et al.^26^ *P* < 5 × 10^−8^) for an association with the height PGS in *n* = 305,485 unrelated individuals of European descent. Of the tested height-associated rare variants, 14 were also associated with the height PGS (at P_FDR_ < 0.05). To determine whether local LD between the rare and common variants in the PGS was driving these associations, we re-derived the PGS, excluding each chromosome from the score calculation. Eleven variants lost their PGS association when the chromosome on which they were carried was removed from the score, indicating these associations were being driven by local LD (Fig. 5a). The remaining three rare variants continued to tag the polygenic background (1:212072099:A:G P_height_ = 2.0 × 10^−8^, P_PGS_ = 0.001, P_PGS_nochr1_ = 0.0008; 3:188609231:C:T P_height_ = 9.5 × 10^−13^, P_PGS_ = 0.007, P_PGS_nochr3_ = 0.006; 6:30147543:G:T P_height_ = 1.3 × 10^−10^, P_PGS_ = 0.0002, P_PGS_nochr6_ = 0.002; Fig. 5a). Notably, one carrier of the ultrarare variant on chromosome 1 (MAF = 0.000008, MAC = 4) had a PGS and standardised height in the 0.01^st^ and 0.2^nd^ percentile of the dataset respectively (Supplementary Fig. 10), making this a candidate variant for confounding by an extreme polygenic phenotype within an extended family. The four carriers of this variant in our dataset were related beyond the third degree (as the cohort had been pruned of related individuals below this threshold), but we were underpowered to determine whether they exhibited greater relatedness than expected by chance.

We then assessed how related rare variant carriers would have to be for a shared variant private to the family, and a shared polygenic background at the extremes of the distribution, to result in false positive associations. Both measured height and polygenic height showed a substantial correlation between individuals related < = 5^th^ degree, after which point an individual’s height, or height PGS, failed to predict that of more distant relatives (Supplementary Fig. 11). Cohort simulations demonstrated that the rate of decay in PGS correlation over generations was related to both trait heritability and assortative mating, and most closely mirrored empirical data for height (where estimates in European cohorts sit at h^2^ ~ 0.66–0.82^31^ and rho ~ 0.26^32^) when h^2^ and rho were set to 0.4 (Fig. 5b). Supplementary Note 2 details simulations we conducted to investigate the liability to familial polygenic confounding of de novo variants transmitted through generations. Supplementary Figs. 12 and 13 show that simulated de novo null rare variants emerging in a lineage with an extreme mean phenotypic value can show substantial correlations with a measured trait or trait PGS (Supplementary Fig. 13) when trait heritability and assortative mating are high, though other unmodelled factors, including sample size and environmental factors, will likely influence the strength of this correlation. This confounding decays rapidly in the generations proceeding a mutational event, though the presence of substantial assortative mating slows this decay. Including the PGS in the analysis model reduced the strength of association with null variants in early generations, but only if the fraction of trait heritability explained was high (Supplementary Fig. 14). Additionally, we assessed whether a truly causal rare variant may associate with a PGS under strong assortative mating, which can induce correlations between all causal variants. Simulations demonstrated that the correlation induced between a PGS and a causal rare variant is negligible, even when assortative mating is extreme (Supplementary Fig. 15). Thus, our findings demonstrate that false positive rare variant associations can arise due to local LD with common causal variants, and our simulations suggest that familial polygenic confounding may result in spurious associations with rare variants private to extended families with extreme phenotypes. In practice, the severity and consequence of the latter mechanism will depend on the degree of relatedness in the study population, and the extent to which phenotypic correlations between distal relatives are preserved by assortative mating or shared environmental factors. We detect evidence of potentially spurious height-associated rare variants, which show strong associations with the common variant height PGS independent of local LD, and promote sensitivity analyses assessing rare variant-PGS associations to flag potentially unreliable rare variant effects confounded by relatedness.

### Biased rare variant effect estimates bias MR analyses

Given the evidence of bias in population effect estimates for rare height-associated variants, we compared the WS to population-based MR estimates of the causal effect of height on educational attainment (EA). Observational associations between height and EA potentially arise through a combination of in-utero and early childhood environment factors, social confounders, assortative mating and dynastic effects^33^. In the UKB, where both height and EA (years in education) are non-uniformly spatially distributed (Fig. 4a, Supplementary Figs. 6, 16), the observational association of measured height with EA, correcting for age and sex, is strongly positive (ß = 0.157; 95% CI 0.153–0.161 s.d. increase in EA per s.d increase in height), and attenuates slightly with inclusion of birthplace coordinate, CTYUA of birthplace, or 150 HCs in the model (coordinate adjustment ß = 0.143; 95% CI 0.138–0.148; CTYUA adjustment ß = 0.130; 95% CI 0.125–0.134; HC adjustment ß = 0.135; 95% CI 0.130–0.140). We used population, and WS estimates for 54 independent rare height-associated variants (MAF < 0.01, MAC > 20, Backman et al.^26^ *P* < 1 × 10^−5^) to instrument height (Supplementary Data 2). Population-based MR estimates supported a positive causal effect of height on education (0.09; 95% CI 0.01–0.18 s.d. increase in EA per s.d increase in height), but the WS estimate was attenuated toward the null (−0.01, 95% CI −0.22–0.20; Supplementary Fig. 17), although the confidence intervals between the two estimates did overlap due to low precision (β_diff_ = 0.1, CI −0.07–0.27, *P* = 0.25). Both population and WS MR estimates using rare genetic instruments were concordant with corresponding estimates derived using common variants^15^ (Table 1). This finding suggests that the mechanisms biasing the causal MR estimate of height on education in population-based analysis using common variants also apply to those using rare variant instruments.

**Table 1:** Inverse variance weighted (IVW) causal effect estimates of a s.d. increase in height on a s.d. increase in years in education, based on rare or common instrumental variables (IVs) for height. 95% confidence intervals are shown

GWAS model	Rare IVs	Common IVs^15^
Population	0.09 (0.01–0.18)	0.06 (0.04–0.07)
Within-sibship	−0.01 (−0.22, 0.20)	0.02 (−0.01, 0.04)

## Discussion

The extent to which direct genetic effects estimated from rare variant GWAS are biased by the factors known to confound common variant studies is unknown. Simulations have illustrated that recent demography, which is poorly corrected for by traditional methods of population stratification control, confounds rare variant associations^16,17^. Using the UKB WES cohort, we have investigated this issue empirically. As anticipated, the distribution of rare variants in the UK reflects an emergent fine-grained population structure^9^, with evidence of allele clustering by birthplace and large frequency differences between proximal counties. Whilst rPCs were more consistent across odd/even chromosomes, and therefore better describe population structure, neither the cPCs nor rPCs generated here, which captured overlapping sources of ancient genomic variation (likely due to LD between variants nearing 1% MAF included in both), could completely control population stratification in a negative-control rare variant GWAS of birthplace, supporting theoretical evidence that PCs are ill-suited to capture fine-grained demography^6,16,17^. It is possible that including whole genome sequencing variants capturing a greater number of independent genomic regions, as well as variants at lower frequency (e.g., <0.01% MAF), would improve rPC performance and minimise redundancy^34^, but the efficacy of including PCs in sequence-based GWAS is likely bounded^23^. Inclusion of millions of ultra-rare and private variants, and large numbers of fitted PCs, would be required to capture better the increasingly granular demography detectable as cohort sizes grow. This is neither computationally resourceful nor productive, as lower-order PCs are liable to tag local genomic structure^28^ or outlying groups of cryptically related individuals. Although not explicitly assessed here, inclusion of rare variants in GRMs has also been shown to degrade performance^23^, adding noise to relationship estimates when using highly lineage-specific markers, which fail to recapitulate more ancient relatedness structure. HCs, and other methods of capturing identity by decent through sharing of genome segments^9^, have proven more efficacious in modelling fine-grained demography^24^. These present a tractable alternative for both rare and common variant GWAS, particularly for stratified traits that continue to show confounding due to residual population structure^35^. However, despite maximally reducing genomic inflation in negative-control GWAS, false-positive associations remained with highly structured rare variants post HC fitting, emphasising that sensitivity analyses that are robust to population stratification will become increasingly important in validating emerging rare variant associations.

Within-sibship re-estimation of rare pLOF associations with quantitative measures^26^ showed minimal effect heterogeneity on a per-variant basis but systematic bias across all variants associated with some traits, including height and red blood cell parameters. We show a general trend that as traits become more spatially clustered, the deviation between population and within-sibship estimates increases, suggesting an underlying issue of inadequately captured population structure. This is not a phenomenon unique to rare variants. Common variant PGS generated for complex traits that align strongly with geography continue to associate with geography upon adjustment for 40 PCs, and show biased trait associations due to residual latent structure^35^. We explored sources of bias in rare variant associations with standing height, which is spatially patterned and exhibits common variant associations confounded by demographic and dynastic effects^11,15^. However, these analyses indicated that systematic bias in rare variant height effects is more likely to be driven by assortative mating, dynastic or other indirect genetic effects, than residual population structure. Both rare and common variant associations can absorb signal from these phenomena, and accordingly, population-based MR estimates of the effects of height on educational attainment showed similar shrinkage in siblings when using either common or rare genetic instruments, despite their differing demographic histories^10,15^. Risk of spatial confounding is likely to be most acute when rare variants and traits show overlapping, highly localised distributions^16,17^. We lacked power in the sibling cohort to assess very rare variant ( < 0.01% MAF) associations with more geographically constrained traits, such as binary health measures influenced by localised environmental risk factors, regional socioeconomic disparities or centre-specific misclassification, which will need to be addressed with more targeted analyses. The availability of geographical data in sequenced biobanks will be important to assess the unique genetic and phenotypic spatial structure of studied populations to inform on the risk of rare variant spatial confounding in future population-based analyses. Assessing the spatial autocorrelation of residuals following PC or HC fitting may help flag traits for which uncorrected spatial structure is liable to confound highly structured rare variants.

We additionally demonstrate that spurious or biased rare variant associations may arise due to LD with highly significant common causal variants, despite showing minimal linkage (Supplementary Note 3). This could be mitigated by including the PGS in analysis models, and this is advised, though efficacy would depend on the fraction of heritability the score captures. Beyond genetic confounding, which also strongly impacts common variant GWAS, we explore the mechanism of familial polygenic confounding, a biasing phenomenon expected to be unique to rare variant studies entirely due to the familial restriction of very rare variants. Conceptually, this issue is akin to, but more acute than, the known issue of cryptic relatedness, though it has been argued that the shared ancestry and environment of related individuals in common variant GWAS is unlikely to induce substantial genotype-phenotype correlation in reality^30^. In GWAS of rare variants, it is possible for a single lineage to carry the entirety of a genetic signal, and every variant private to an extended family at the extreme tails of the PGS distribution would be susceptible to strong confounding due to what is essentially an issue of sharp population structure. Such confounding would also be expected in situations where an extreme phenotype arises through a shared environment (the most acute example of the spatial confounding explored above), though environmental effects would feasibly be less specifically restricted to dispersed distant relatives than is the inheritance of a shared polygenic background. Through simulations, we show that when null rare variants emerge within a family with a high polygenic burden, underlying an extreme phenotype, they correlate strongly with polygenic trait liability and trait expression, which takes multiple generations to decay when heritability and assortative mating are high. This correlation is greater than that expected between a causal rare variant and the polygenic component of a trait due to assortative mating, such that identifying strong rare variant associations with the PGS (with the target chromosome excluded) presents a practical means to distinguish unreliable results. Common null variants are unlikely to be biased by this mechanism due to being widely distributed across a population of diverse genetic backgrounds. Population stratification could theoretically also induce rare variant-PGS correlations, which these sensitivity analyses would also detect.

We found three height-associated rare variants, which are also associated with the height PGS independent of local LD, which we reasoned may present evidence for familial polygenic confounding. One such ultra-rare variant was carried by an individual with an extreme outlying height and height PGS, though we were unable to confirm whether all carriers of this variant exhibited higher than expected relatedness. Though further empirical evidence is required to assess the precise scenarios in which such confounding may arise, it is important to consider, as a driver of false positives, as the rapid growth of sequenced population cohorts facilitates further rare variant association studies. Increasing the threshold for relatedness in GWAS beyond the standard >3^rd^ degree may alleviate the risk of familial confounding, though it would come at the expense of sample size. Although fitting of rare variant derived GRMs within LMMs may appear a natural solution to this problem, private variants would be expected to contribute minimally to their construction, and genetic relatedness calculated genome-wide would not necessarily capture the higher regional relatedness between individuals sharing a rare variant by decent at a specific genomic locus. Newly developed methods to construct ancestral recombination graphs (such as Relate^36^ and ARG-Needle^37^) rely on rare variants for the explicit inference of recent, locus-specific ancestry, and may allow family-level confounding to be better addressed by fitting genealogy-informed local GRMs alongside global GRMs in LMMs. Finally, the prioritisation of family-based recruitment into future sequencing studies for within-family analyses will remain a reliable solution to assess the robustness of rare variant estimates to complex confounding mechanisms^38^.

This work is limited to focusing on only a small subset of rare, damaging coding variants with significant complex trait associations. Nevertheless, we show that demographic confounding factors influencing common variants may also bias the estimation of direct rare variant genetic effects, and demonstrate the unsuitability of common and rare variant-derived PC in controlling for these. We have not explored bias in gene-based burden tests, which aggregate across rare variants with differing confounding structures, and predict these to be more robust to the confounding mechanisms described^17^. Although small systematic biases may not substantially alter the biological interpretation of genome-wide significant rare variant associations, confounding factors acting genome-wide may have a large aggregate effect on multi-variant methods such as MR, heritability, polygenic score and genetic correlation calculations. Further, we consider acute familial polygenic confounding as a mechanism with the potential to induce strongly confounded single rare variant association with private variants in outlying families, which will likely require more effective correction for granular relatedness structure to address. Overall, methods to more effectively capture fine-grained population structure are expected to improve the accuracy of population-based rare variant analyses, and judicious use of family-based methods will remain an important approach to assessing the reliability of emergent rare variant associations into the future.

## Methods

### Ethics

Individual-level data and summary statistics were taken from the UK Biobank (UKB), a UK population cohort recruited from the general population aged 40 to 69 years residing in the UK between the years of 2006 and 2010. Participants provided written informed consent for collected physiological, anthropometric, sociodemographic and biological (including genetic) data and linked healthcare records, to be used for research purposes. Ethical approval was granted by the NHS National Research Ethics Service North West (16/NW/0274), and data was accessed under approved Application ID 81499. Full details of the UKB are provided online: https://www.ukbiobank.ac.uk/about-our-data/.

### Sample selection

Genotype, whole-exome sequencing and phenotypic data for 502,357 UK Biobank (UKB) participants were accessed through the UK Biobank DNANexus Research Analysis Platform. First-degree siblings were identified from the available participant relatedness file by thresholding on kinship coefficient (>=0.117, <0.354) and IBS0 (proportion of SNPs sharing zero alleles) >0.001. Individuals related to another participant to the third degree or greater were excluded from population-level analyses. Ancestry inference was performed with KING^39^ by projecting UKB samples onto the 1000 Genomes Project ancestry reference set, using 144,443 independent genotyped variants. A total of 38,601 siblings and 306,991 unrelated individuals of European ancestry with >90% genotype call rate across whole-exome sequenced rare variants (described below) were used in downstream analyses.

### Principal component generation and haplotype components

#### Common variant PCs

Common variant principal components were generated with the pcapred.largedata package^40,41^ by projecting European samples onto pre-derived PCs from a UKB reference population, using a shared set of 147,604 genotyped autosomal variants in linkage equilibrium. Odd and even chromosome-derived PCs were generated using flashpcaR^42^ by first extracting variants on odd chromosomes (*n* = 74,651) and even chromosomes (*n* = 72,953) independently from the pcapred reference SNP set.

#### Rare variant PCs

Rare variant PCs were generated with flashpcaR^42^ for unrelated European samples, using a total set of 617,375 WES-derived SNPs at MAF < 1%, MAC > ~50, pruned to exclude linked variants at *r*^*2*^ > 0.1 within each 10 mb window (with 2 mb step size). WES variants failing the 90pct10dp filter described in the UKB 500k WES processing documentation (requiring 90% of genotypes at a given variant to have a read depth of at least 10) were also excluded. Odd and even PCs were generated independently using odd (*n* = 342,418) and even (*n* = 274,957) chromosome-derived SNPs. Rare PCs for the sibling cohort were obtained by projecting siblings onto the reference flashpca object generated for unrelated samples above using the equivalent SNP set.

#### HCs

Haplotype components were generated for UKB individuals using PBWTpaint, as described by Yang et al. in the associated manuscript^24^. Briefly, genome-wide pairwise haplotype sharing (coancestry) was estimated by painting each UKB individual against all others using 569,242 phased genetic variants. This produces a sparse matrix in which each element represents the total length of haplotype segments an individual copies from another. This matrix was log-transformed and decomposed using singular value decomposition (SVD), and the top 150 components (from $U\sqrt{D}$) were retained as haplotype principal components (HCs).

### Within-sibship model

#### Phenotype and variant selection

We reassessed rare variant associations with complex traits reported by Backman et al.^26^ using a within-sibship (WS) model. Single variant tests of putative loss of function (pLOF) and deleterious missense coding region variants (SNPs and indels) reaching the reported study-wide threshold of significance for rare variants (*P *< 2.18 × 10^−11^) in European participants were retrieved from published supplementary data table SD2. UKB field IDs for tested traits were used to extract corresponding sibling phenotypic measures. Quantitative traits (including anthropomorphic, biochemical and haematological measures) only were carried forward to downstream analysis due to the difficulty of precisely recapitulating phenotype definitions for ICD10-based binary disease traits and low case numbers in the sibling subset. Quantitative phenotypes collected across repeat visits were averaged per participant, and the final set of 155 measures were inverse rank normalised. The same phenotypes were also extracted from the full set of unrelated participants to assess trends in spatial clustering as described below. Rare variants ( < 1% MAF) reaching a single-trait threshold of suggestive association (*P* < 1×10^−5^) were identified from the published GWAS summary statistics accompanying the Backman et al.^26^ study for the 155 phenotypes above. Genotype dosages (0,1 or 2 counts of the minor allele) for the required variants were extracted from the PLINK-formatted genotype files for siblings. Variant effect estimates for 1939 variant-trait pairs were re-estimated in siblings using the model below.

#### Analysis model

Only sibships with non-missing phenotype, genotype and covariate data for a given variant-trait combination were used in each analysis, with an average of *n* = 26,679 (range 2123–37,057) individuals analysed per trait. The statistical model to derive WS estimates of variant effects was applied to the sibling cohort using linear regression in R, as described in Howe et al.^15^. Briefly, the mean family genotype ${G}_{i}^{F}$ for each sibship $i$ was calculated over $n$ siblings and used to centre each individual’s genotype around the sibship mean; ${G}_{{ij}}^{C}$, centred genotype of sibling $j$ in sibship $i$. For example, in a family (family 1) where sibling 1 is a heterozygous carrier of a rare variant and sibling 2 is homozygous for the major allele ${G}_{1}^{F}=\frac{0+1}{2}=0.5$, ${G}_{{{\mathrm{1,1}}}}^{C}=1-0.5=0.5$ and ${G}_{{{\mathrm{1,2}}}}^{C}=0-0.5=-0.5$. Centred genotype was regressed against phenotype with correction for mean family genotype, age, sex, age^2^, age x sex, 10 common and 20 rare principal components, as included by Backman et al.^26^:

1 $$	{{Trait}}_{{ij}} \sim {G}_{{ij}}^{C}+{G}_{i}^{F}+{{Age}}_{{ij}}+{{Sex}}_{{ij}}+{{Age}}_{{ij}}^{2}+{{Age}}_{{ij}}\times {{Sex}}_{{ij}}+{{PC}1}_{{ij}}^{{common}} \\ 	+\ldots+{{PC}10}_{{ij}}^{{common}}+{{PC}1}_{{ij}}^{{rare}}+\ldots+{{PC}20}_{{ij}}^{{rare}}$$

where ${G}_{i}^{F}=\frac{{\sum }_{1}^{n}{G}_{{ij}}}{n}$ and ${G}_{{ij}}^{C}={G}_{{ij}}-{G}_{i}^{F}$ with ${G}_{{ij}}$ being the genotype (minor allele count) of sibling *j* in sibship *i*. Standard errors were clustered over sibships by extracting parameter estimates from the variance-covariance matrix using vcovCL from the package sandwich^43^, and using coeftest from lmtest^44^ to derive clustered SE for the variant effect and p-values. Results for variant-trait pairs where fewer than 20 minor alleles had been observed in the sibling set were removed from downstream analysis.

### Polygenic height score

The polygenic score (PGS) for adult standing height was constructed using 3,198 genome-wide significant variants associated with height, weighted by their conditional effect estimates, from the Genetic Investigation of Anthropometric Traits (GIANT) consortium interim GWAS meta-analysis of height (excluding 23andMe and UK Biobank, N = 1,400,860 European individuals)^45^. The PGS for UK Biobank participants was computed using PLINK2^46^ based on hard-call genotype data. Leave-one-out scores were re-derived with each chromosome excluded in turn. Rare variant associations with the derived scores calculated using the --fastGWA-lr function from GCTA^19^ with correction for 10 common and 20 rare PCs.

### Statistical analysis

All analyses were performed using custom R (v4.4.0) scripts and publicly available packages.

#### Assessing PCs

Pearson’s correlation between odd and even chromosome-derived PCs was calculated for PCs1-20 in turn, for both rare and common PC sets. Variant loadings across each PC were extracted to assess chromosome-specific contributions to each component. Canonical correlation analysis was used to assess the variance in 1) odd PCs captured by even PCs and vice versa, 2) common PCs captured by rare PCs and vice versa, and 3) rare PCs captured by HCs and vice versa. The cancor function from the package candisc^47^ was used to find orthogonal linear combinations of each PC set having the maximal canonical correlation. The redundancy function was used to derive the proportion of the variance captured by the first set of PCs (or HCs) accounted for by the second through the canonical variates. To assess the extent to which the first 30 rare PCs could predict each of the first 30 HCs, and vice versa, we calculated the coefficient of determination (R^2^) from a series of linear models in the form:

2 $${HC}1 \sim {{PC}1}_{{rare}}+\ldots+{{PC}30}_{{rare}}$$

The same approach was used to assess the variation in the 155 measured traits (plus the coordinate of birthplace and years of education) explained by the first 30 rare PCs or the first 30 HCs.

#### GWAS of birthplace

To assess the utility of PCs and HCs in correcting for population stratification in rare variant genetic association studies, a GWAS of the north coordinate of birthplace (UKB field ID 129) was performed in 279,390 unrelated individuals of European ancestry born in the UK. Linear regression was run using the --fastGWA-lr function from GCTA^19^, with inclusion of variable combinations of common and rare variant derived PCs (no PCs, PC1-2 rare, PC1-5 rare, PC1-10 rare, PC1-20 rare, PC1-2 common, PC1-5 common, PC1-10 common, PC1-20 common, PC1-10 common and PC1-10 rare, PC 1-10 common and PC1-20 rare, PC1-10 common and PC1-30 rare, PC1-20 common and PC1-30 rare, PC1-40 common and PC1-30 rare), 30 or 150 HCs. Genomic inflation factors (GIF; λ_GC_) were calculated as the median of resulting chi-squared test statistics for a given model, divided by the expected median of the chi-squared distribution. GIFs were compared across models of varying PC inclusion, and calculated for variants at differing minor allele frequency thresholds ( < 0.01%, 0.01–0.1%, 0.1–1%), as well as between variants that did or did not exhibit significant spatial autocorrelation as determined by Moran’s I statistic (calculated for chromosome 1 variants only; see Statistical Analysis below), post maximal PC inclusion (PC1-40 common and PC1-30 rare).

#### Comparing within-sibship and population model estimates

Restricting to the subset of functional (pLOF or deleterious missense) coding rare variants showing associations with 155 quantitative traits (at *P* < 1 × 10^−5^) in the Backman et al. study^26^, we compared published population-level effect estimates to the estimates returned from the WS model. WS estimates were regressed against population estimates for 1939 variant-trait pairs to obtain the regression slope. It was assessed whether this slope differed significantly from 1 (the null hypothesis of $\beta=1$ indicating perfect concordance between model estimates) by performing a two-tailed *t*-test where:

3 $$t=\frac{{\hat{\beta }}_{1}-1}{{{SE}}_{{\hat{\beta }}_{1}}}$$

Analysis was repeated for each trait independently for those traits with at least 8 associated variants, with and without restricting to the top associated variant per gene when necessary. Cochran’s Q test was used to test for heterogeneity in single variant-trait estimates between models.

#### Variant and trait spatial clustering

It was hypothesised that rare variants exhibiting substantial non-uniformity in their spatial distribution (e.g., restricted to a narrow geographical range) may be more susceptible to confounding by demographic factors in rare-variant GWAS. To quantify spatial autocorrelation of both rare variants and measured traits, individuals were first assigned to a county or unitary authority of birth using northing and easting coordinates of birthplace (UKB field IDs 129 and 130), based on the December 2023 ultra-generalised UK vector boundaries from the Office for National Statistics^48^, using the st_intersects function from the R package sf^49^. Neighbouring area polygons were defined according to the provided shapefile with the poly2nb function from the package spdep^50^, assigning equal weights to neighbours. The minor allele frequency per area was derived for each variant, and Moran’s I statistic was calculated using the moran.test function. Moran’s I statistic captures the extent to which neighbouring areas have similar values of a feature of interest, ranging between −1 (dispersed) and 1 (clustered) through 0 (randomly distributed). Variants showing clustering with a false discovery rate-adjusted *p*-value of <0.05 were considered significantly spatially autocorrelated. The same approach was used to calculate Moran’s I statistic for the studied quantitative measures. The average distance between the birthplaces of variant carriers was taken as the average pairwise Euclidean distance between variant carriers in kilometres and tested for an association with log_10_(MAF) using linear regression. The association between the trait’s Moran’s I statistic and the change in slope estimated by regressing WS against population derived rare variant trait effect estimates was assessed by linear regression, weighting by the inverse squared standard error of the slope estimate:

4 $${lm}(\left|\beta -1\right| \sim {Morans}\,I,\,{weight}=\frac{1}{{{SE}}^{2}})$$

Where β and SE were taken from the linear model *lm(population* ~ *WS)*, using SNP effect estimates for trait associated variants, as described above.

#### Comparing within-sibship and area model estimates

Rare variant effects on height (UKB field ID 50) for 42 rare height-associated variants identified in Backman et al.^26^ (P < 5 × 10^−8^ and MAC > 20) were re-estimated in 279,391 unrelated European individuals born in the UK using two area-informed models. Model 1 tested for within and between area effects by regressing an individual’s area-centred genotype ${G}_{{ai}}^{C}$ on rank normalised standing height, correcting for mean area genotype ${G}_{a}^{A}$, where ${G}_{a}^{A}=\frac{{\sum }_{1}^{n}{G}_{{ai}}}{n}$ and ${G}_{{ai}}^{C}={G}_{{ai}}-{G}_{a}^{A}$ with ${G}_{{ai}}$ being the genotype (minor allele count) of individual *i* in area *a* and *n* being the number of individuals born in the area (akin to the sibling model but with individuals grouped by CTYUA of birth rather than sibship):

5 $${{Height}}_{{ai}} \sim {G}_{{ai}}^{C}+{G}_{a}^{A}+{{Age}}_{{ai}}+{{Sex}}_{{ai}}+{{Age}}_{{ai}}^{2}+{{Age}}_{{ai}}\times {{Sex}}_{{ai}} \\+{{PC}1}_{{ai}}^{{common}}+\ldots+{{PC}10}_{{ai}}^{{common}}+{{PC}1}_{{ai}}^{{rare}}+\ldots+{{PC}20}_{{ai}}^{{rare}}$$

Model 2 included northing and easting coordinates of birthplace in a standard genetic association model:

6 $$	{{Height}}_{i} \sim {G}_{i}+{{{Northing}}_{i}+{{Easting}}_{i}+\,{Age}}_{i}+{{Sex}}_{i}+{{Age}}_{i}^{2}+{{Age}}_{i}\times {{Sex}}_{i} \\ 	+{{PC}1}_{i}^{{common}}+\ldots+{{PC}10}_{i}^{{common}}+{{PC}1}_{i}^{{rare}}+\ldots+{{PC}20}_{i}^{{rare}}$$

Where ${G}_{i}$ is the genotype (minor allele count) of individual *i*. In a third model, the first 150 HCs were fitted to capture fine-grained ancestry:

7 $${{Height}}_{i} \sim {G}_{i}+{{Age}}_{i}+{{Sex}}_{i}+{{Age}}_{i}^{2}+{{Age}}_{i}\times {{Sex}}_{i}+{{HC}1}_{i}+\ldots+{{HC}150}_{i}$$

Models 2 and 3 were also fitted with the genotype terms removed to derive residual height following correction for included covariates. The Moran’s I statistic of the returned residuals was calculated using moran.test as previously.

#### Mendelian randomisation

The causal effect of height on years in education was estimated using 90 independent, rare (MAF < 0.01) height-associated variants (*P* < 1 × 10^−5^) from Backman et al.^26^ as genetic instruments. Variant effect estimates for the exposure and outcome variables were taken from either the original population GWAS^26^ (height only) or the WS model with clustered SE described above. As population GWAS rare variant associations with the outcome variable (years in education) were not published by Backman et al.^26^, these were calculated using an equivalent GWAS model with internally derived rare and common variant PCs in 303,980 unrelated European individuals from the UKB with –-fastGWA-lr from GCTA^19^. The years in education phenotype was taken from the UKB education level data field p6138, scaled as per the International Standard Classification of Education (ISCED) definitions as follows: 20 = “College or University degree”, 19 = “NVQ or HND or HNC equivalent”, 15 = “Other professional qualification”, 13 = “A levels/AS levels or equivalent”, 10 = “O levels/GCSE or equivalent”, 10 = “CSEs or equivalent”, 7 = “None of the above”, and rank normalised. The observational association between height and educational attainment was assessed using a linear or linear mixed effects model with the inclusion of age and sex as covariates, or the addition of northing and easting coordinate or birthplace, 150 HCs, or a random effect term of birth CTYUA with varying intercept.

The mr function from the TwoSampleMR^51^ package was used to derive the inverse variance weighted (IVW) causal effect estimate for the effect of a SD increase in height on a SD change in years in education using each of the population and WS-derived instrument effects for exposure and outcome variables in turn. Steiger testing and visual inspection of per-variant Wald ratios were used to confirm that all instruments explained more of the variance in the outcome variable than the exposure, and to check for outlying SNP effects. Heterogeneity in population and WS-derived IVW estimates was assessed using the difference of two means test (ß_IVW,diff_ = ß_IVW,pop_- ß_IVW,WS_) with jack-knifing to estimate the standard error of the difference for P-value calculation, as described in Howe et al^15^. Rare variant-derived IVW estimates were compared to published common variant estimates for the same exposure-outcome relationship^15^.

### PGS and trait correlations between related individuals

Genetic relatedness was calculated for a randomly down-sampled set of 100,000 UKB European participants with the --make-grm function from GCTA, using ~100,000 genotyped autosomal variants in linkage equilibrium (filtered using PLINK2 --indep 2000 500 1.05). Pairs of individuals related to the n^th^ degree were extracted by filtering based on relatedness coefficient (1^st^:0.375–0.75 2^nd^:0.188–90.375, 3^rd^:0.0938–0.188, 4^th^:0.0469–0.0938, 5^th^:0.0234–0.0469, 6^th^:0.0177–0.0234, 7^th^:0.00586–0.0117, 8^th^:0.00195–0.00586, unrelated:<0.00195), ensuring each individual was represented in only one pair within each relatedness group. Groups were further down-sampled to approximately equal size (*n* ~ 775), and the Pearson’s correlation between standardised measured height and height PGS in each was calculated. For details of simulations, see Supplementary Note 2.

### Reporting summary

Further information on research design is available in the Nature Portfolio Reporting Summary linked to this article.

## Supplementary information

Supplementary Information

Description of Additional Supplementary Files

Supplementary Dataset 1

Supplementary Dataset 2

Reporting Summary

Transparent Peer Review file

## Source data

Source Data

## Data Availability

UK Biobank population exome summary statistics for complex traits are provided as supplemental data with the publication by Backman et al.^26^ and have been deposited within the GWAS Catalogue [https://www.ebi.ac.uk/gwas/]; see Backman et al.^26^ Supplementary Data Table 4 [https://www.nature.com/articles/s41586-021-04103-z#Sec18] for all study accession numbers. Within-sibship model summary statistics for top-associated rare variants are available in Supplementary Data 1 with GWAS Catalogue accession numbers linking to full summary statistics. Individual-level genetic and phenotypic data for UK Biobank participants are accessible by approved researchers; for more information on data access, visit https://www.ukbiobank.ac.uk/use-our-data/. Area boundaries used for mapping UK counties and unitary authorities were taken from the December 2023 ultra-generalised UK vector boundaries from the Office for National Statistics [https://geoportal.statistics.gov.uk/datasets/countries-december-2023-boundaries-uk-buc/about]. Source data for figures is provided with this paper within a source data file. Source data are provided with this paper.

## Code availability

Analysis scripts used to generate the results reported here are publicly available through GitHub [https://github.com/alhbiostat/rare-variant-bias.git] and on Zenodo [10.5281/zenodo.19558747]. Rare variant polygenic confounding simulations included in the Supplementary Notes are available on Github [https://github.com/alhbiostat/rare-polygenic] and Zenodo [10.5281/zenodo.19558818].
