# Supplementary material for: Evaluating confounding in rare variant genome wide association studies
Source: Nat Commun. 2026 May 29;17:7002. doi: 10.1038/s41467-026-73776-9 (PMC13392019; doi:10.1038/s41467-026-73776-9)
Supplement: Supplementary file 1 — Supplementary Information [file 41467_2026_73776_MOESM1_ESM.pdf]

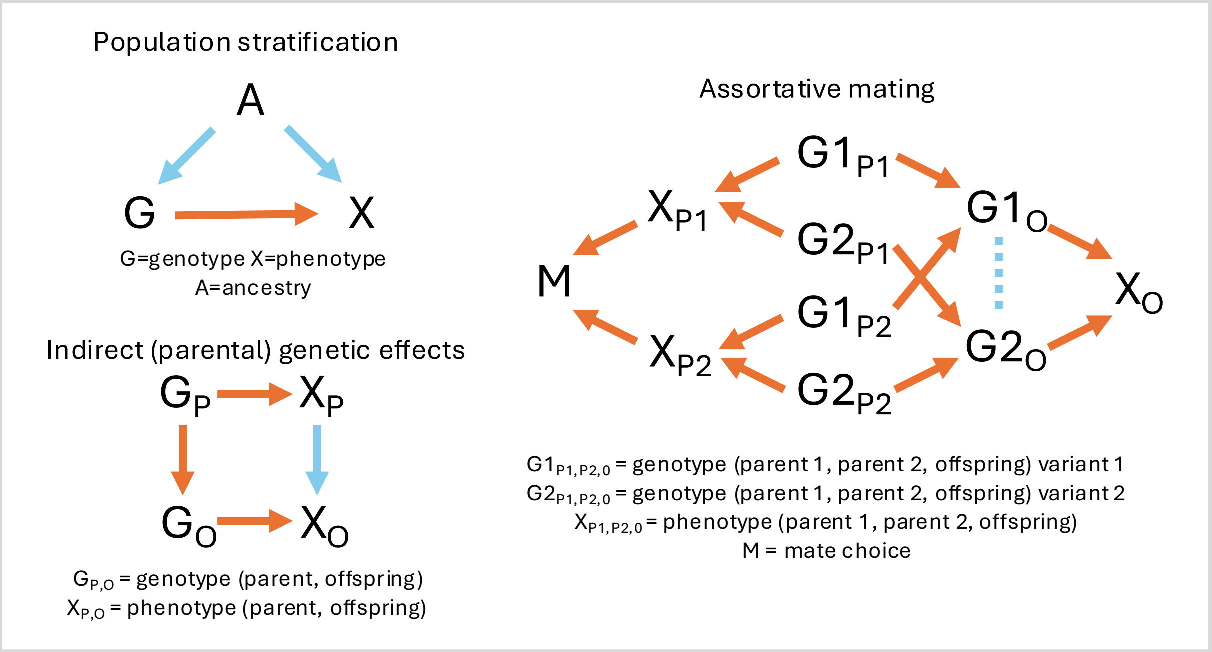

Supplementary Figure 1: The three primary mechanisms biasing direct genetic effect estimates from population genome wide association studies. Population stratification; the confounding of genotype-phenotype associations by ancestral differences across a study population, which is associated both with variation in allele frequencies, and sub-population phenotypic variation due to spatial/cultural/societal factors. Assortative mating; the correlation of independent loci contributing to a phenotype influencing mate choice in the parental generation in offspring. Independent loci subsequently absorb the genetic contribution of phenotype associated loci genome wide, distorting trait associations. Indirect genetic effects; the contribution of parental genotype to offspring phenotype both via direct allele transmission and modification of the shared environment through parental phenotype. Indirect effects acting through other relatives (e.g. siblings) are also possible, but potentially less influential.

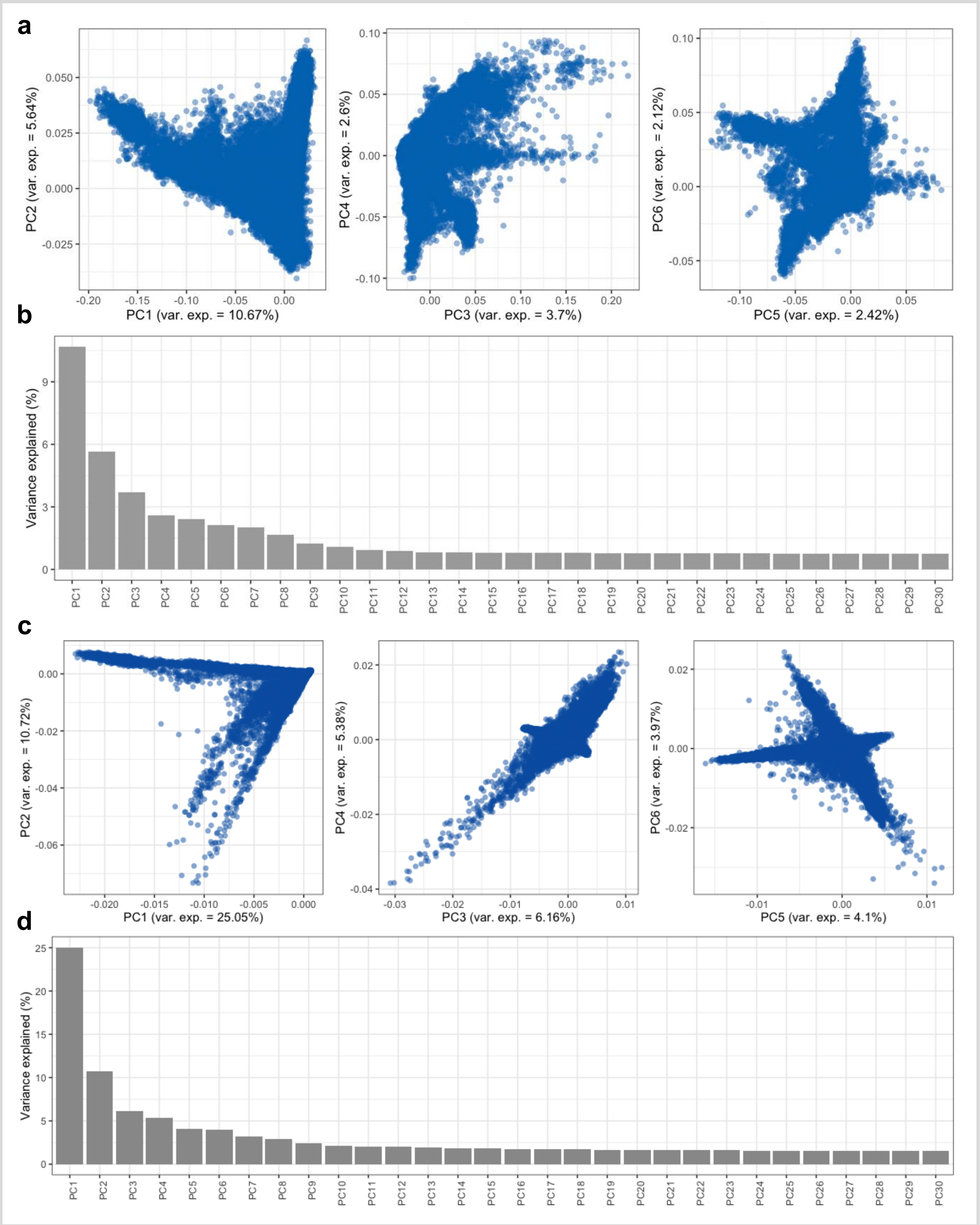

Supplementary Figure 2: Component plots showing PCs1-6 and scree plots of percent variance explained for the first 30 principal components generated using 147,604 independent common SNPs (MAF >1%) (a,b) and 617,375 rare SNPs (MAF < 1%, MAC > 50) (c,d) for 306,991 individuals of European decent.

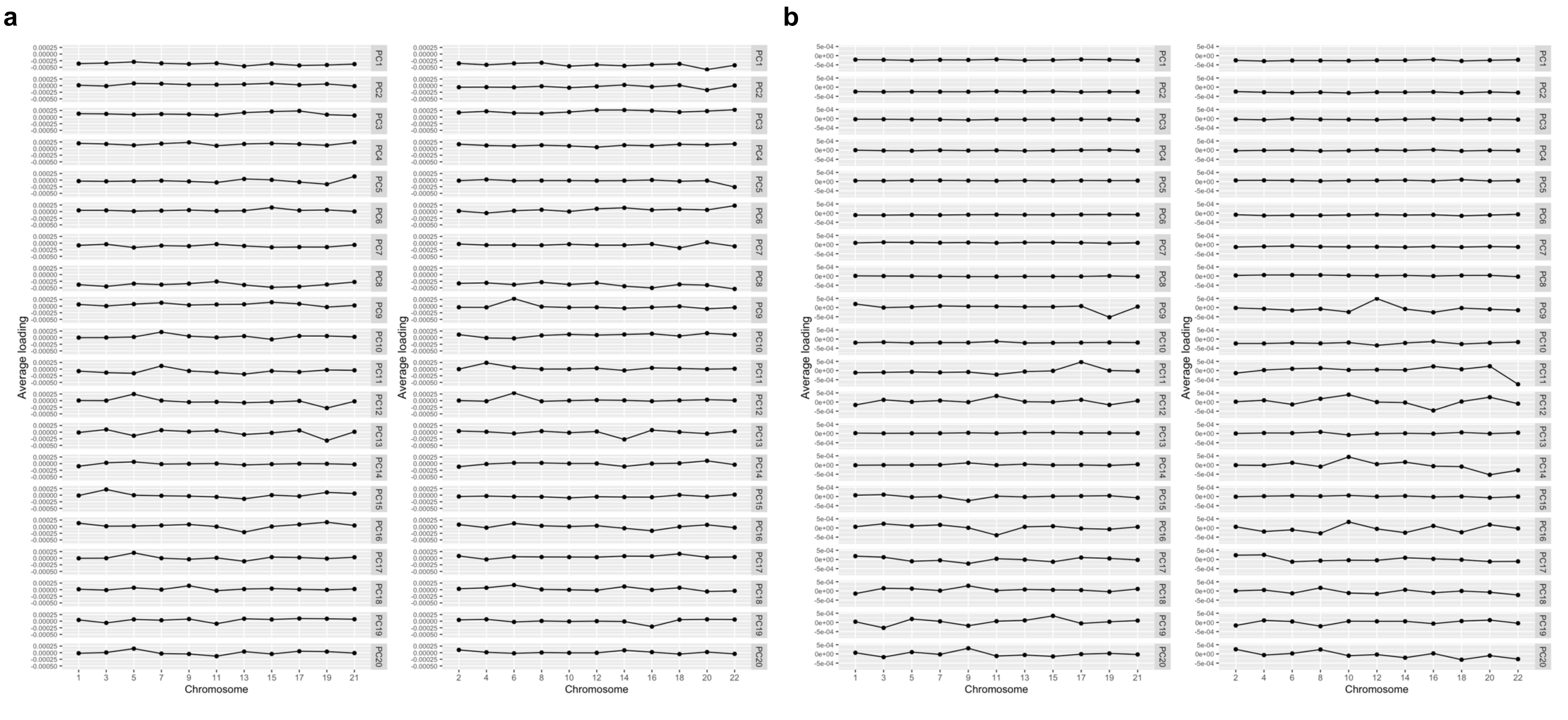

Supplementary Figure 3: Average loadings for variants across odd and even chromosomes for the first 20 common (a) and rare (b) variant derived PCs.

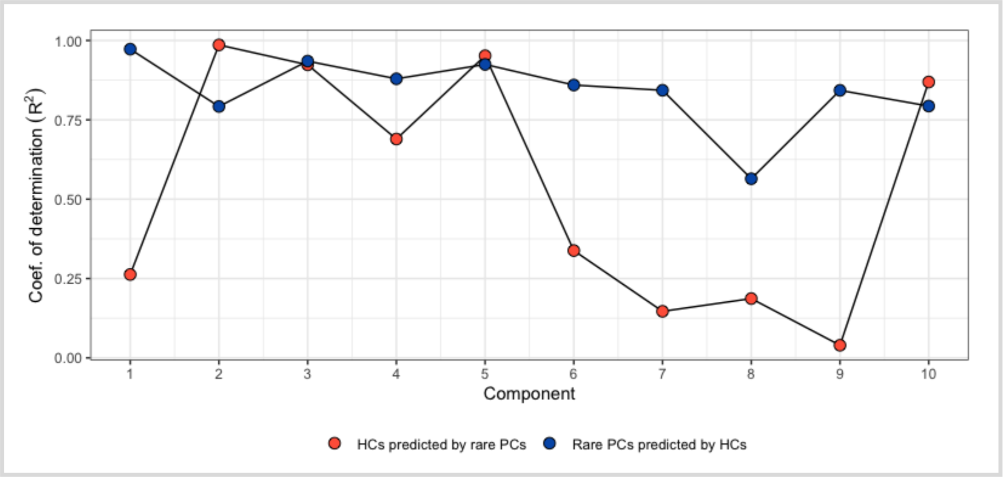

Supplementary Figure 4: Coefficient of determination (R^2) for the prediction of the first 10 rare variant derived principal components (PCs) from the first 30 haplotype components (HCs) when fitted in a linear model, and vice versa.

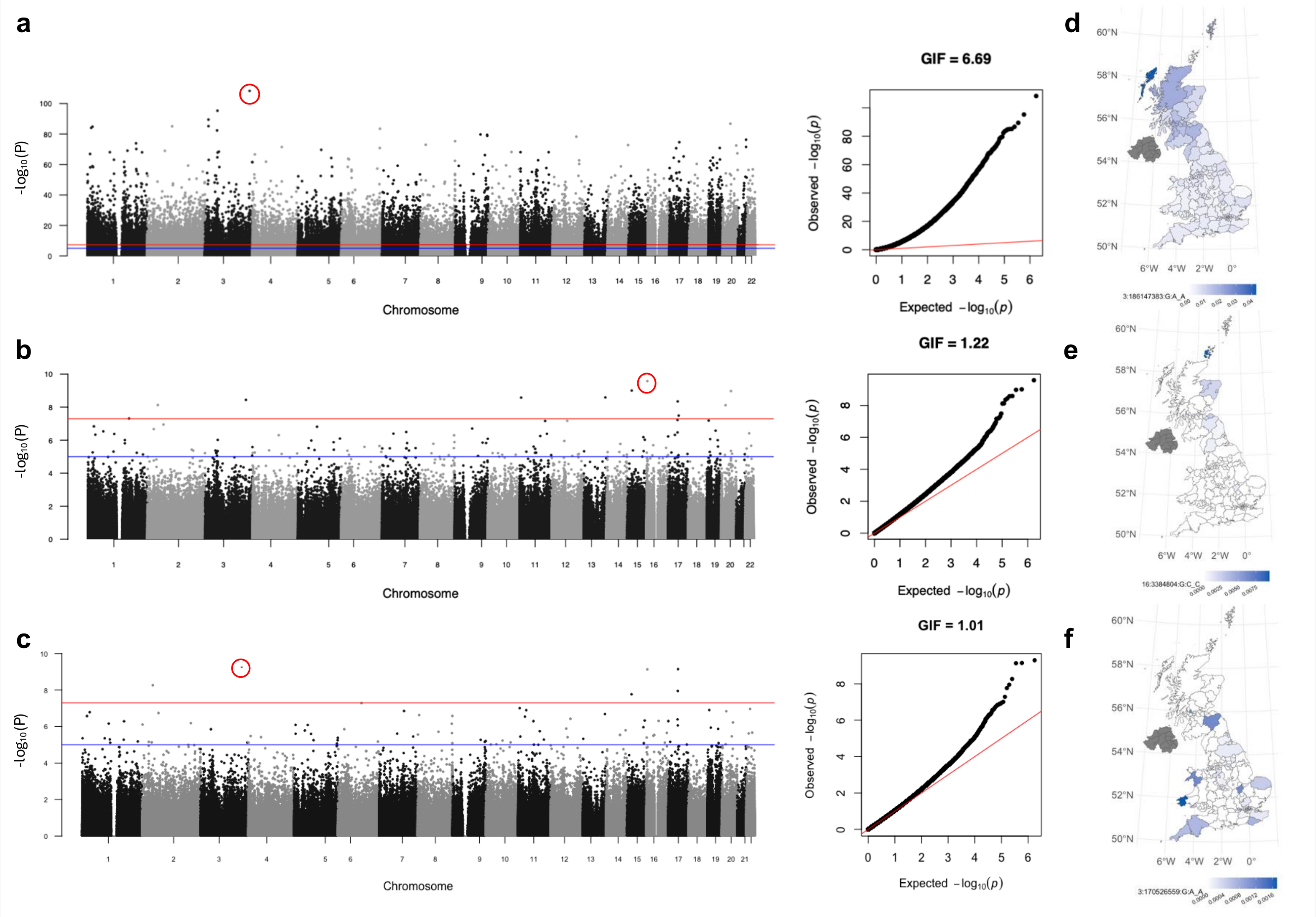

Supplementary Figure 5: Manhattan plot and accompanying Q-Q plot with genomic inflation factor (GIF) for the rare variant GWAS of north co-ordinate of birthplace in 279,390 UK born individuals of European ancestry, (a) without population stratification correction, (b) with inclusion of 40 common and 30 rare variant derived principal components and (c) with inclusion of 30 haplotype components. The geographical distribution of the top birthplace associated variant in each analysis (circled in red) is shown in (d-f) respectively. Minor allele frequencies are averaged across county or unitary authority of birth.

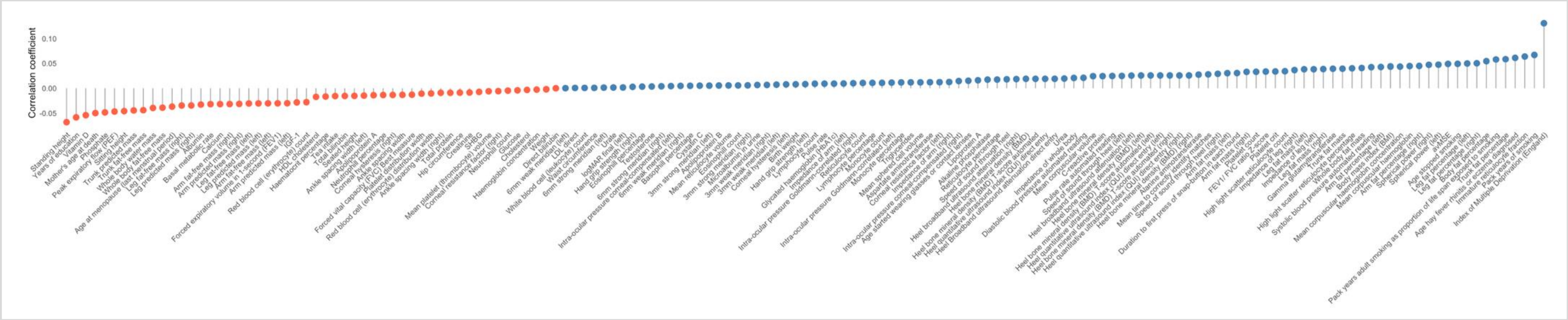

Supplementary Figure 6: Pearson correlation between 163 quantitative traits and north coordinate of birthplace in 279,390 individuals of European decent born in the UK. All measures have been inverse rank normalised. Negative correlations are shown in red, and positive correlations in blue.

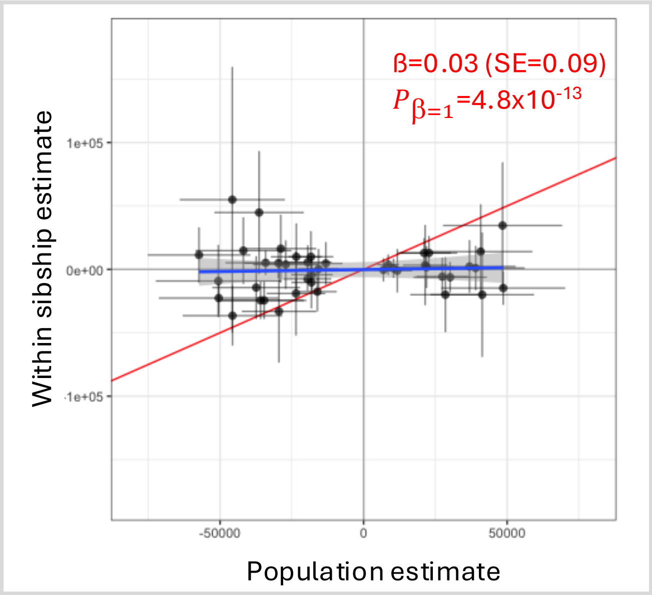

Supplementary Figure 7: Regression of variant effect estimates from population GWAS (with 10 common and 20 rare PCs fitted as covariates) against those from a within-sibship model, for 42 rare variants associated with northing coordinate of birthplace (population $P<1\times10^{-5}$). P-value is provided for the two-tail t-test of difference in slope from 1 (red line). Bars represent 95% confidence intervals.

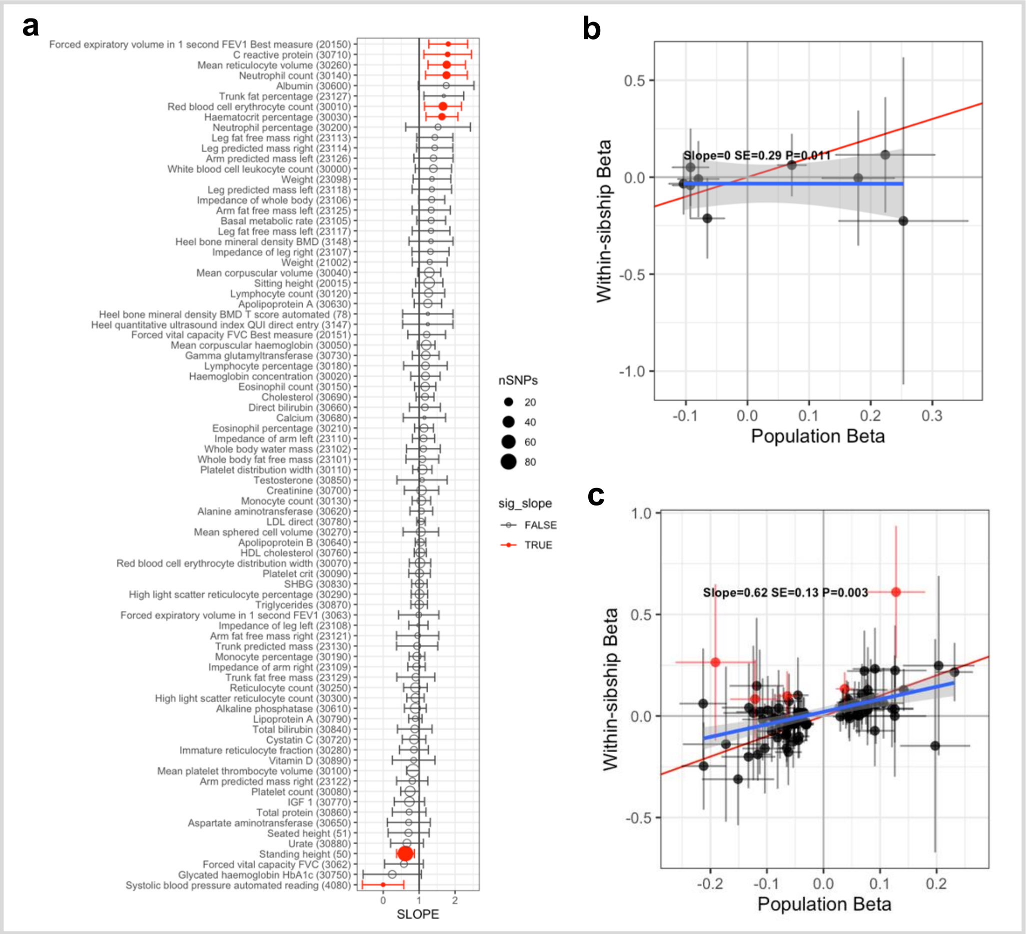

Supplementary Figure 8: Slope of regression line fitted between population GWAS and within-sibship model effect estimates for trait-associated (population $P<1\times10^{-5}$) rare variants, retaining the top variant per gene. (a) Slope estimates across all quantitative traits, with those significantly differing from 1 at $P<0.05$ coloured red. Regression of variant effect estimates from both models are shown for systolic blood pressure (b) and standing height (c); variants coloured red show significant heterogeneity between models. Significance is prior to multiple testing correction. Bars represent 95% confidence intervals.

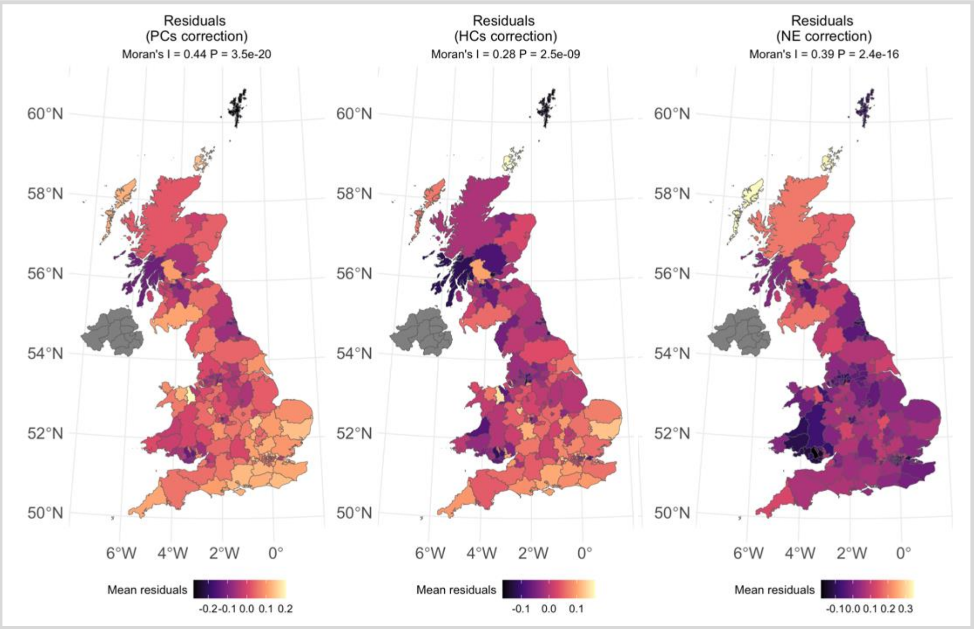

Supplementary Figure 9: Spatial distribution of residual height following correction for 10 common and 20 rare PCs, 150 HCs or northing and easting coordinate of birthplace. Individuals are grouped by county or unitary authority (CTYUA) of birthplace based on the December 2023 Office of National Statistics ultra-generalised UK vector boundaries. The mean of residuals is calculated per CTYUA boundary and used to calculate of the Moran's I statistic of spatial autocorrelation.

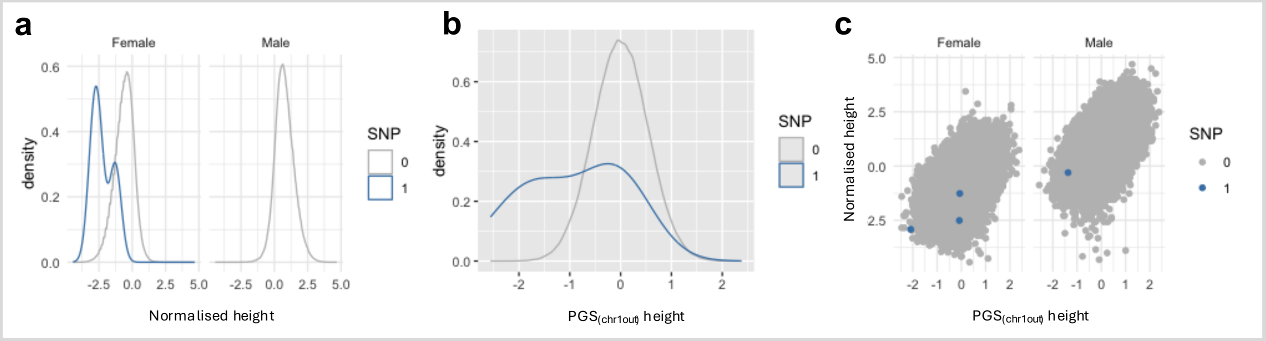

Supplementary Figure 10: (a) Distribution of rank normalised height in carriers (n=4) and non-carriers (n= 305,485) of the 1:212072099 rare G allele (MAC = 4). No data shown for the single male carrier. (b) Distribution of the standardised height PGS (with chromosome 1 removed; residuals after regressing against 10 common and 20 rare PCs) in rare variant carriers and non carriers. (c) Scatter plot of residualised, standardised height PGS (with chromosome 1 removed) against measured height in male and female rare variant carriers.

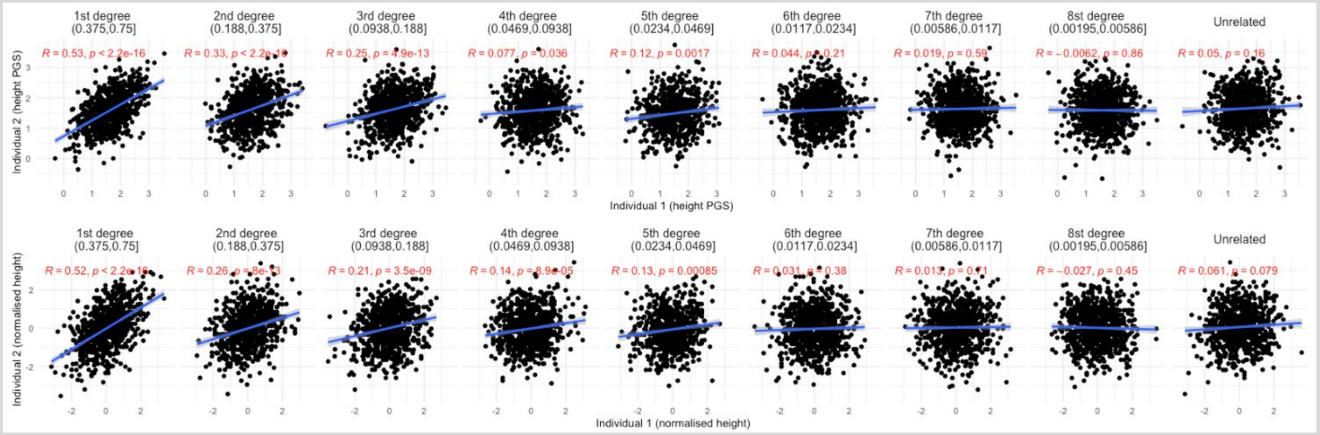

Supplementary Figure 11: Correlation between height PGS (top) and normalised measured height (bottom) in pairs of UKB European individuals related to the nth degree (n~775 per relatedness group).

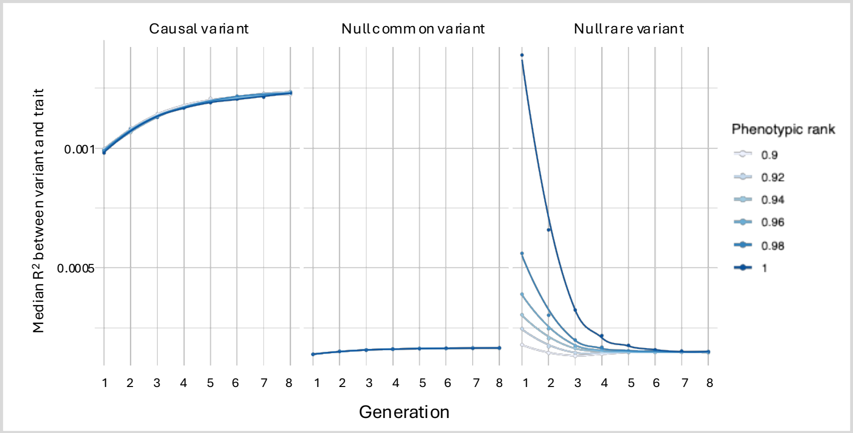

Supplementary Figure 12: Median variance explained (R^2) in polygenic trait by simulated causal common variants (n=500), null common variants (n=1000) and null rare variants (n=10000) over eight successive generations. Founder individuals were assigned common variant genotypes generated under a binomial distribution with varying allele frequencies, a polygenic score given as the weighted sum of alleles at n=500 causal loci, and a phenotype calculated using the derived PGS and an environmental contribution scaled by an assigned trait heritability (h^2) of 0.8. Causal rare variants were set as heterozygous in the founder mating pair with a phenotypic value at differing phenotypic ranks. Pairs were matched by phenotype to induce phenotypic correlation ρ=0.4. Subsequent generations receive randomly transmitted alleles at each loci.

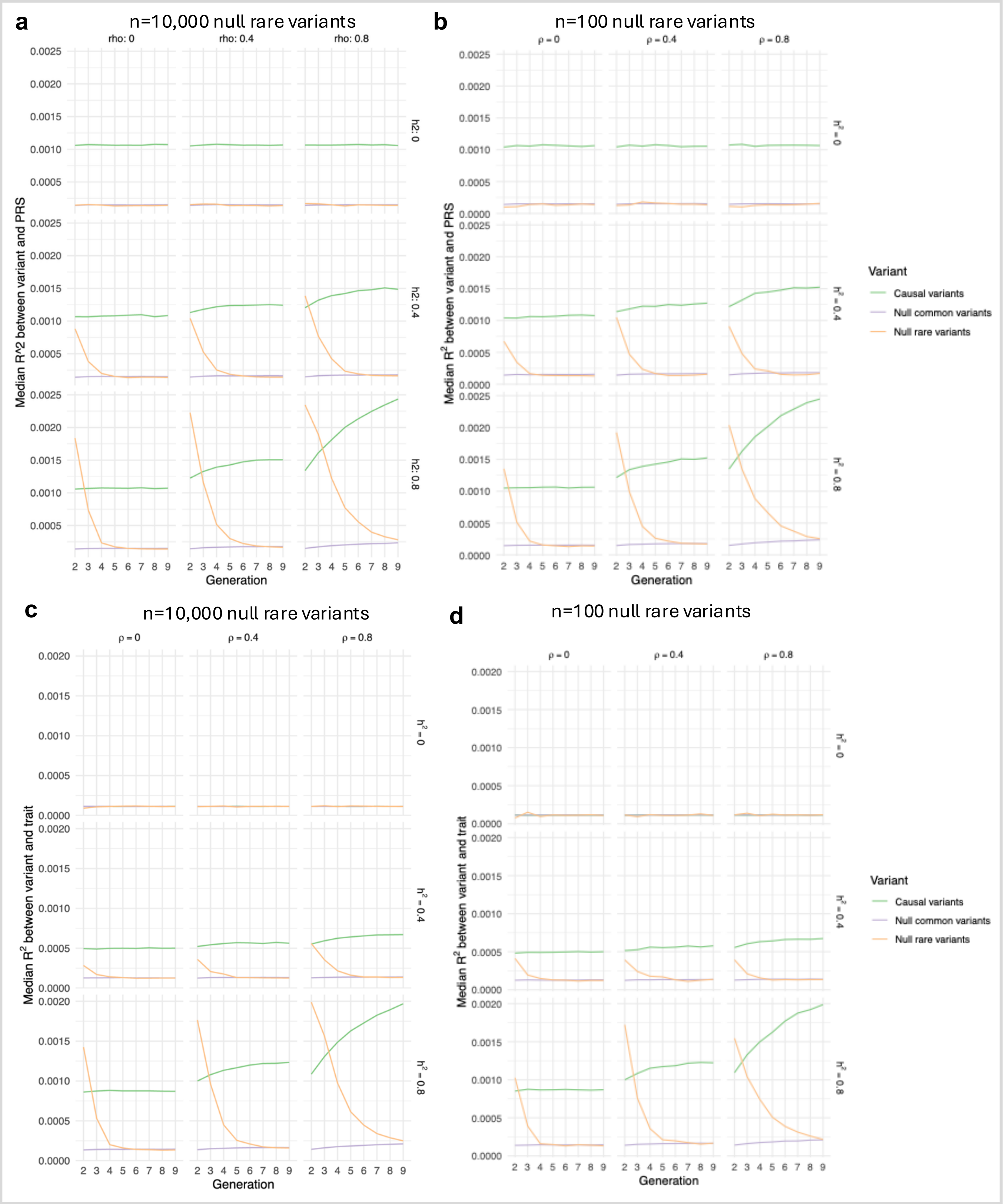

Supplementary Figure 13: Median variance explained (R^2) in derived common variant PRS (a and b) or the derived trait value (c and d) by simulated causal common variants (n=500; green), null common variants (n=1000; purple) and null rare variants (n=10000 (a and c) or n = 1000 (b and d); orange) over ten successive generations, under varying trait heritability (h^2) and assortative mating (ρ) . Founder individuals were assigned common variant genotypes generated under a binomial distribution with varying allele frequencies, a polygenic score given as the weighted sum of alleles at n=500 causal loci, and a phenotype calculated using the derived PGS and an environmental contribution scaled by an assigned trait heritability (h^2). Null rare variants were set as heterozygous in the founder mating pair with the highest phenotypic value. Pairs were matched by phenotype to induce phenotypic correlation ρ. Subsequent generations receive randomly transmitted alleles at each loci.

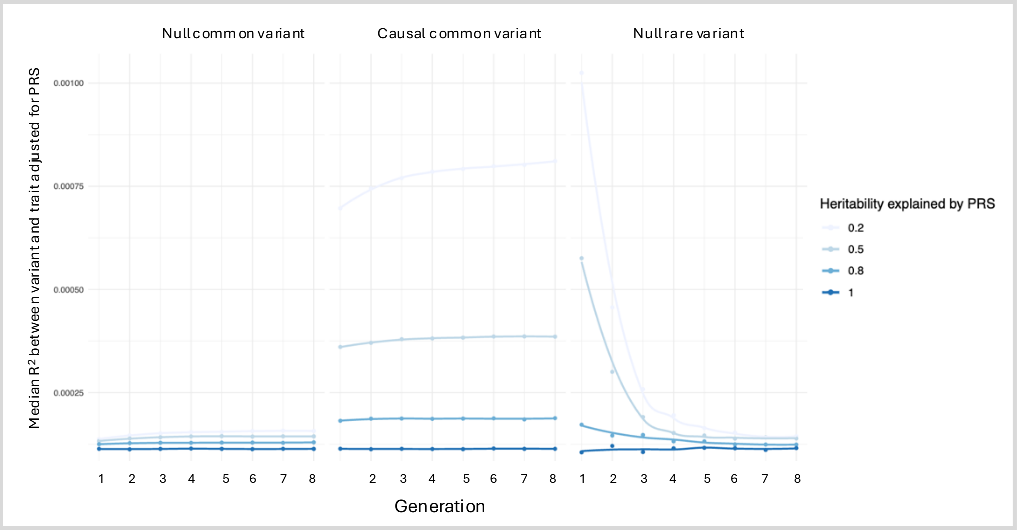

Supplementary Figure 14: Median variance explained (R^2) in a polygenic trait by simulated null common variants (n=1000, left), causal common variants in the PRS (n=500, middle), or null rare variants (n=10,000, right) over eight successive generations when a PRS capturing differing fractions of trait heritability are included in the model. Trait heritability (h^2) was fixed at 0.8 and assortative mating (ρ) at 0.4. Founder individuals were assigned common variant genotypes generated under a binomial distribution with varying allele frequencies, a polygenic score given as the weighted sum of alleles at n=500 causal loci, and a phenotype calculated using the derived PGS and an environmental contribution scaled by an assigned trait heritability (h^2). Null rare variants were set as heterozygous in the founder mating pair with the highest phenotypic value. Pairs were matched by phenotype to induce phenotypic correlation ρ. Subsequent generations receive randomly transmitted alleles at each loci.

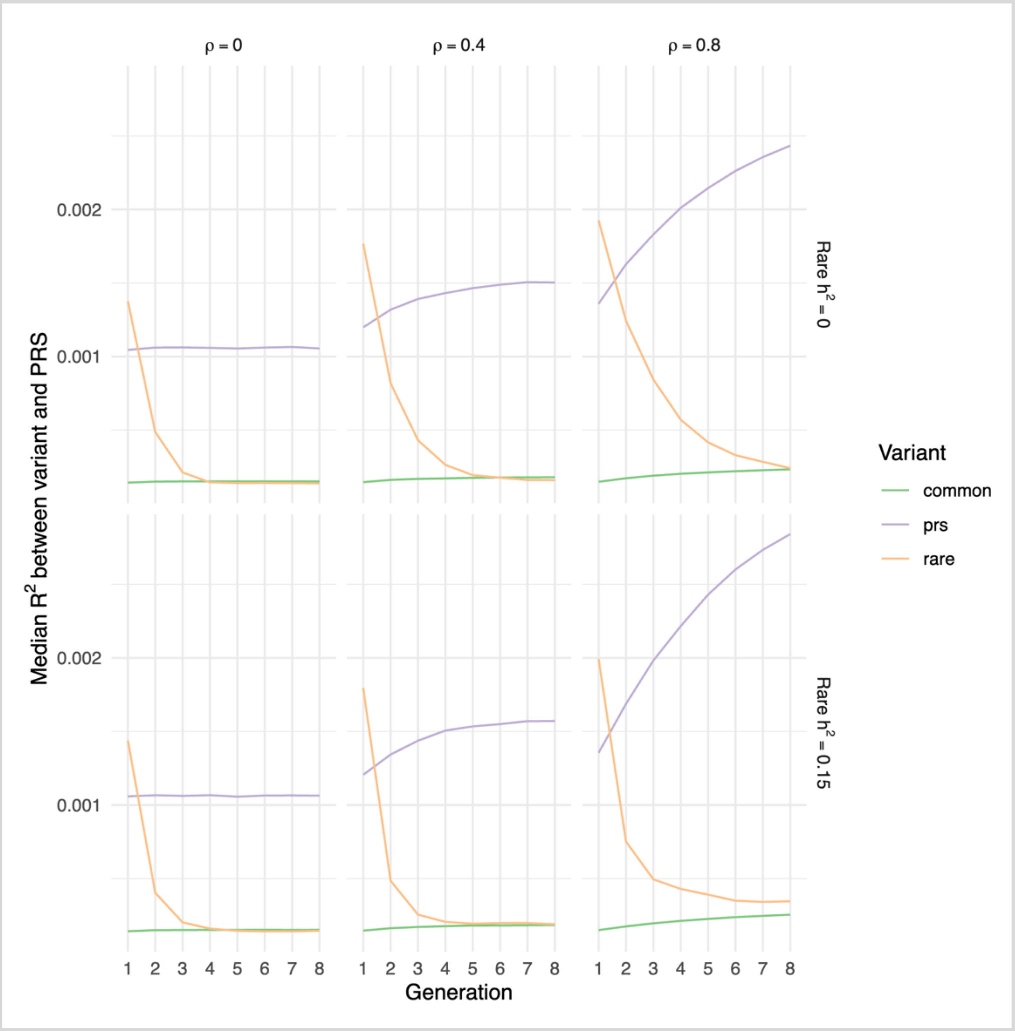

Supplementary Figure 15: Median variance explained (R^2) in derived common variant PRS by simulated causal common variants in the PRS (n=500; purple), null common variants (n=1000; green) and null (top) or causal (bottom) rare variants (n=10000; orange, explaining 0 or 15% of total heritability respectively), over eight successive generations, under varying assortative mating (ρ) . Founder individuals were assigned common variant genotypes generated under a binomial distribution with varying allele frequencies, a polygenic score given as the weighted sum of alleles at n=500 causal loci, and a phenotype calculated using the derived PGS and an environmental contribution scaled by an assigned trait heritability (h^2=0.8). Null or causal rare variants were set as heterozygous in the founder mating pair with the highest phenotypic value. Pairs were matched by phenotype to induce phenotypic correlation ρ. Subsequent generations receive randomly transmitted alleles at each loci.

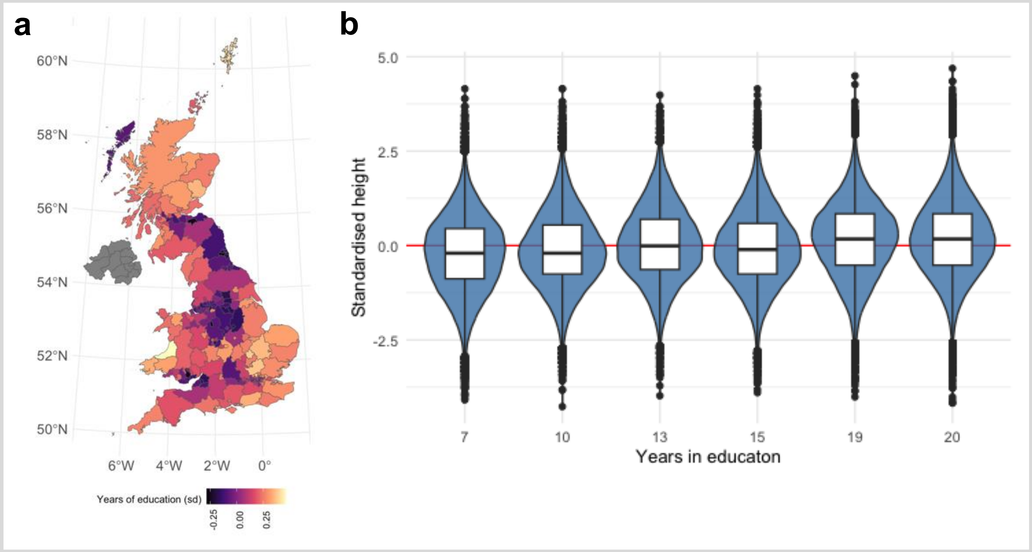

Supplementary Figure 16: (a) Spatial distribution of rank normalised years in education (derived from UKB field p6138). Individuals are grouped by county or unitary authority (CTYUA) of birthplace based on the December 2023 Office of National Statistics ultra-generalised UK vector boundaries. The mean measure is calculated per CTYUA boundary and used to calculate of the Moran's I statistic of spatial autocorrelation. (b) Violin plots showing distribution of standardised height in individuals split by years in education.

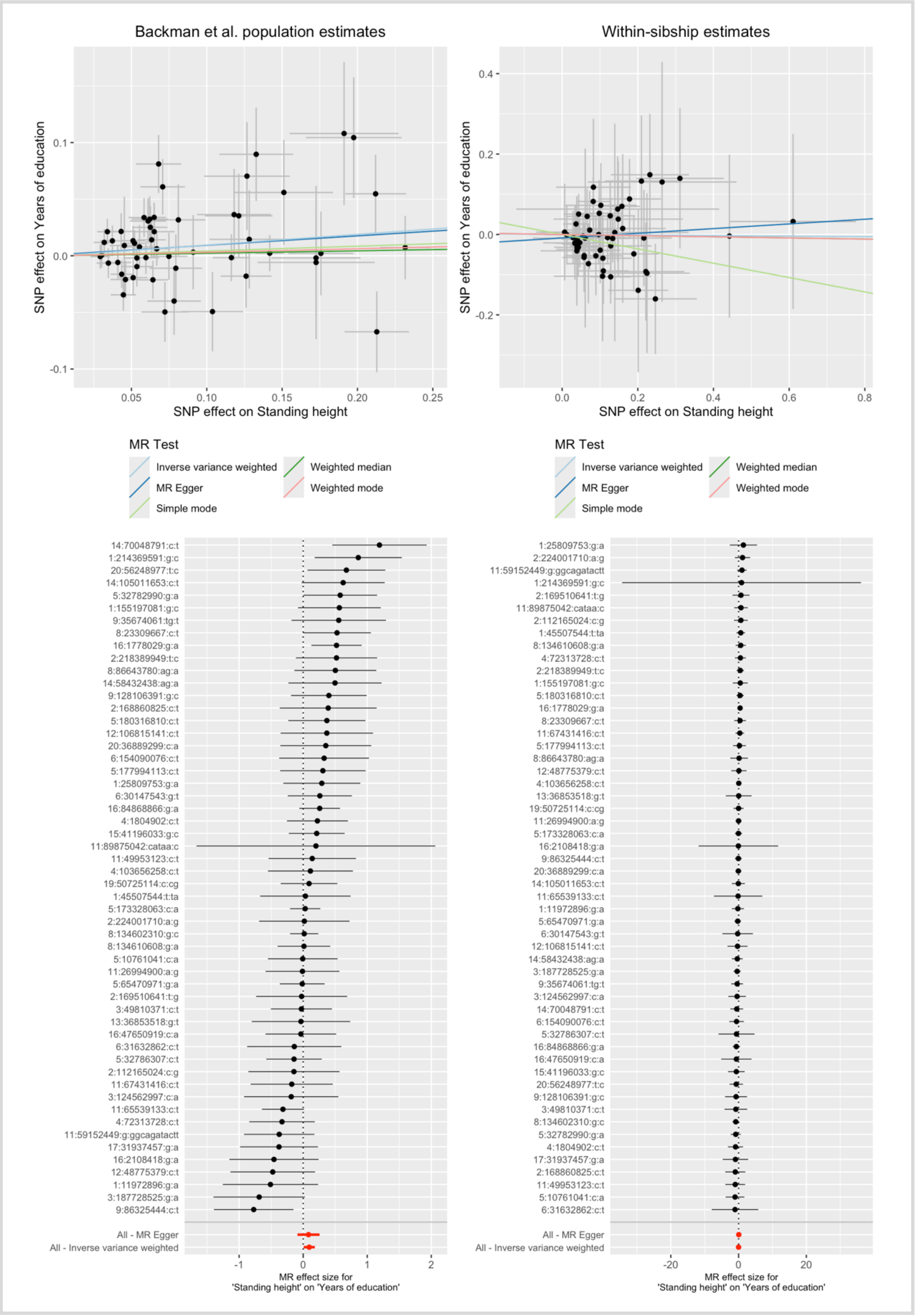

Supplementary Figure 17: Rare variant Mendelian randomisation analysis of the causal effect of height on years in education using population (left) or within-sibship (right) derived effect estimates for exposure and outcome variables. Forest plots show per-variant Wald ratios. Instruments were selected from Backman et al. rare variants (MAF <1%) with height associations at $P<1\times10^{-5}$ and MAC>20 in the sibling cohort.

Supplementary Note 1 – Assessing expected shrinkage in within-sibship estimates under assortative mating

We wished to assess the extent to which shrinkage or inflation of within sibship (WS) rare variant effect estimates for the traits assessed in Fig. 3b could be explained by assortative mating (AM). We followed the approach presented by Lee et al.[1] and Yengo et al.[2], whereby the expected shrinkage across within-family derived variant effect estimates in the absence of population stratification, but in the presence of AM can be given as: $E[S_{PS}] = 1 - rh^2$ (where r = the spousal correlation reached after many generations of assortative mating and h^2 is the narrow sense heritability). We identified n=47,266 spousal pairs in the UKB using the method developed by Howe et al.[3], and calculated spousal correlations using rank normalised phenotype data. In the absence of narrow sense heritability estimates for all traits, we took SNP-based heritability estimates available in the UKB SNP-Heritability Browser (https://nealelab.github.io/UKBB_ldsc/index.html) from the Neale Lab. As SNP heritability estimates typically underestimate narrow sense heritability by ~50%, we approximated h^2 as twice the SNP-based estimate. Expected shrinkage under assortative mating was derived using the equation above, and compared to the observed shrinkage in WS estimates, taken as the slope of the regression of WS on population effect estimates for trait associated rare variants (at $P<1\times10^{-5}$ in Backman et al.[4]). The estimate shrinkage in height effects ascribed to assortive mating was 0.77 (using an estimated $r = 0.24$ and $h^2 = 0.97$), which approximated the shrinkage observed in the WS model (β=0.71), indicating that inflation in the population effect estimates were approximately as expected under AM. Conversely, shrinkage in WS variant effects on platelet counts, and inflation in WS variant effects on a number of other traits related to body composition, red blood cell parameters and C-reactive protein were more extreme than expected under AM, suggesting confounding by additional or alternate mechanisms in population GWAS (**Supplementary Fig. 18**).

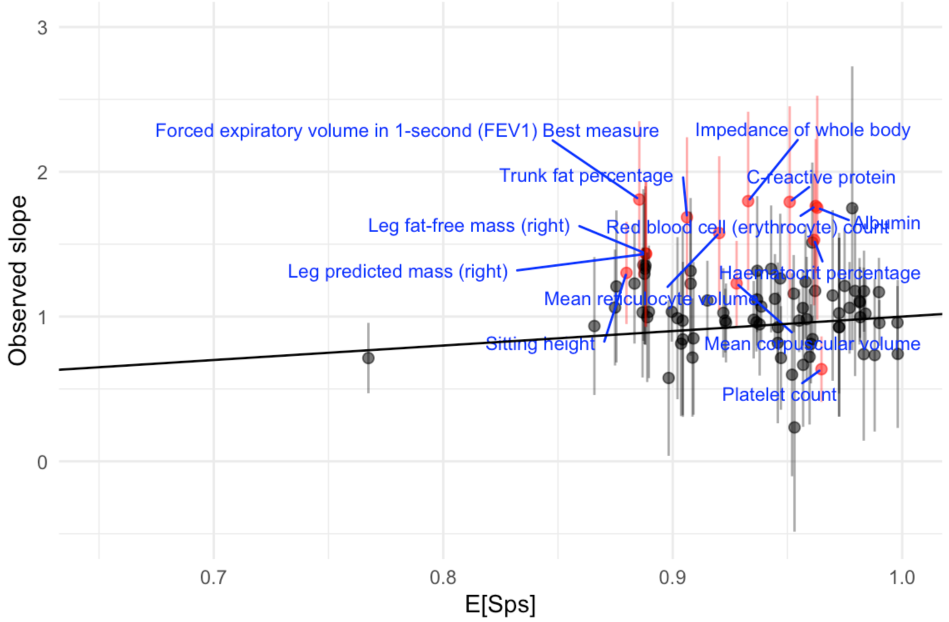

Supplementary Figure 18: Observed vs. expected shrinkage in rare variant effect estimated under assortative mating for 38 complex traits with >=8 rare variant associations at $P<1\times10^{-5}$. For traits labelled and coloured red observed slopes from the regression of WS on population effect estimates does not overlap with the expectation in the absence of population stratification but presence of assortative mating.

Supplementary Note 2 – Assessing liability of rare variants to familial polygenic confounding

We conducted simulations to investigate how polygenic confounding could induce trait associations with non-causal variants, when a family has an excess of alleles at a particular variant compared to the population. That family also shares a similar polygenic profile. We performed forward-in-time individual-level simulations to evaluate the extent to which this phenomenon is possible.

The data-generating model consists of several parts. A founder population is simulated by generating $2n$ individuals, 50% male. Each individual has $m = m_r + m_c + m_p$ null rare, null common and causal common variants. Common variants are generated as $g_{ij} \sim Binom(2, p_j)$ where p_j is the allele frequency for that variant. Rare variants are set to homozygous wild type for all individuals and one of the founding mating pair with the highest phenotypic value were assigned to be heterozygote. Each individual has a polygenic score

$$s_i = \sum^{m_p} \beta_j g_{ij}$$

where $\beta_j \sim N(0,1)$ and a phenotype

$$y_i = \frac{h^2(s_i - \bar{s})}{\sigma_s} + \epsilon_i$$

where $\epsilon_i \sim N(0, (1 - h^2))$.

Founder individuals are paired based on an assortative mating coefficient ρ by simulating a multivariate normal distribution for two variables of length n and with correlation ρ. Vector 1 represents the ordering for the males' phenotypes, and vector 2 represents the ordering for the female phenotypes. The values of y_i are matched based on this ordering to induce a phenotypic correlation between matched males and females of value ρ. Each male-female pair generates two offspring in the next generation by randomly selecting allele transmission at each locus, and then each offspring has a polygenic score and phenotype created in the same manner as the founders. To generate over successive generations, the current generation is randomly paired based on the same value ρ. At each generation, the correlation between each variant and the PRS, and each variant and the phenotype was recorded. Simulations were conducted enumerating the following parameters: $m_p = 500$, $h^2 = (0, 0.4, 0.8)$, $\rho = (0, 0.4, 0.8)$, $m_r = 100 \text{ or } 10{,}000$, $m_c = 1{,}000$, and $n = 2{,}000$. Each simulation was repeated 100 times over 10 generations. The `R/kinship2` package was used to estimate the kinships between individuals in the simulation, which were then used to evaluate the PRS correlations stratified by degree of relatedness.

These simulations were extended in two ways. First, to examine the extent to which correlations between the PRS and the rare variants may be induced when the rare variants are causal. Here, the rare variants were set to contribute an additional 15% of the heritability. Second, to examine the importance of the rare variant carrier's phenotypic rank. We re-ran the simulations where the carrier family had rank at the quantiles $(0.9, 0.92, \ldots, 1)$. Symmetrical results were found when the family rank was at the low end of the scale, where having a rank of 0 led to the highest bias (results not shown).

Supplementary Note 3 – Examining strength of rare variant associations expected due to weak LD between with common causal variants in the PGS

Linkage disequilibrium between two variants is known to be limited by the difference in the allele frequency of the two variants. For a rare variant x and a common causal variant g the LD $R^2_{xg} = \frac{D^2}{p_x(1-p_x)p_g(1-p_g)}$ where $D = p_{xg} - p_x p_g$. The maximum possible LD is $\max\,(R^2_{xg}) = \frac{p_x - p_x p_g}{p_x(1-p_x)p_g(1-p_g)}$ where all rare alleles are on a single haplotype background. Therefore the maximum variance explained in a trait y will be $\max(R^2_{xy}) = \max(R^2_{xg})\,R^2_{gy}$. The pwr.r.test function in the R/pwr package can then be used to estimate the statistical power of association for the rare variant assuming it is in maximum LD with the common variant. To examine the extent to which weak LD between common causal variants and rare variants could drive spurious associations at the rare variants, we used the allele frequencies and R^2_{gy} values from 772 independent variants discovered for height in the UK Biobank GWAS[5], and calculated what fraction of those variants were liable to give rise to a rare variant association with statistical power $\geq 80\%$.

We found that with a sample size of 450000 and $\alpha = 5e^{-8}$, 1.3% of the 772 common variants would be liable to give rise to spurious rare variant associations with power $\geq 80\%$ when the rare variant frequency was at 0.01, and 0.5% of the common variants would lead to rare variant associations when the rare variant frequency was at 0.005. The code to conduct these analyses are available here: https://github.com/MRCIEU/rare-polygenic/blob/main/scripts/ld.r.

This is likely to be a slightly conservative simplification of the problem (meaning that the spurious rare variant associations may be a bigger problem than estimated here). This is because the haplotype background for null rare variants may include multiple common causal alleles, especially if the rare variant is relatively recent and is transmitted on a longer haplotype.

SUPPLEMENTARY REFERENCES

1 Lee, J. J. *et al.* Gene discovery and polygenic prediction from a genome-wide association study of educational attainment in 1.1 million individuals. *Nat Genet* **50**, 1112-1121, doi:10.1038/s41588-018-0147-3 (2018).
2 Yengo, L. *et al.* A saturated map of common genetic variants associated with human height. *Nature* **610**, 704-712, doi:10.1038/s41586-022-05275-y (2022).
3 Howe, L. J. *et al.* Genetic evidence for assortative mating on alcohol consumption in the UK Biobank. *Nature Communications* **10**, doi:10.1038/s41467-019-12424-x (2019).
4 Backman, J. D. *et al.* Exome sequencing and analysis of 454,787 UK Biobank participants. *Nature* **599**, 628-634, doi:10.1038/s41586-021-04103-z (2021).
5 Elsworth, B. L. *et al.* MRC IEU UK Biobank GWAS pipeline version 2 (2019).
