# Supplementary material for: Evaluating confounding in rare variant genome wide association studies
Source: Nat Commun. 2026 May 29;17:7002. doi: 10.1038/s41467-026-73776-9 (PMC13392019; doi:10.1038/s41467-026-73776-9)
Supplement: Supplementary file 2 — Description of Additional Supplementary Files [file 41467_2026_73776_MOESM2_ESM.pdf]

Description of Additional Supplementary Files

File: Supplementary Data 1
Description: Within-sibship and population (Backman et al.) GWAS model results for 1939 quantitative trait associated rare variants (MAF <1%, MAC >20 in siblings, P <1x10^-5 in Backman et al.). CHR = chromosome, BP = base pair position, EA = effect allele, OA = other allele, EAF = effect allele frequency, SE = standard error, CI = 95% confidence interval, P = p-value, Q = Cochran's Q statistic, Adj. P = (FDR adjusted p-value). Population (pop) data is taken from Backman et al. Supplementary Data Table SD2.

File: Supplementary Data 2
Description: Genetic instruments used for rare variants Mendelian Randomization analysis of height (exposure) on years in education (outcome) with population (Backman et al.) or within-sibship GWAS derived effect estimates.
CHR = chromosome, BP = base pair position, EA = effect allele, OA = other allele, EAF = effect allele frequency, SE = standard error, P = p-value. Population data is taken from Backman et al. Supplementary Data Table SD2.
