# Supplementary material for: Evaluating confounding in rare variant genome wide association studies
Source: Nat Commun. 2026 May 29;17:7002. doi: 10.1038/s41467-026-73776-9 (PMC13392019; doi:10.1038/s41467-026-73776-9)
Supplement: Supplementary file 5 — Reporting Summary [file 41467_2026_73776_MOESM5_ESM.pdf]

nature portfolio

Corresponding author(s): Aimee Hanson, Gibran Hemani

Last updated by author(s): 14/04/2026

Reporting Summary

Nature Portfolio wishes to improve the reproducibility of the work that we publish. This form provides structure for consistency and transparency in reporting. For further information on Nature Portfolio policies, see our Editorial Policies and the Editorial Policy Checklist.

Statistics

For all statistical analyses, confirm that the following items are present in the figure legend, table legend, main text, or Methods section.

n/a	Confirmed	
☐	☒	The exact sample size (*n*) for each experimental group/condition, given as a discrete number and unit of measurement
☒	☐	A statement on whether measurements were taken from distinct samples or whether the same sample was measured repeatedly
☐	☒	The statistical test(s) used AND whether they are one- or two-sided<br>*Only common tests should be described solely by name; describe more complex techniques in the Methods section.*
☐	☒	A description of all covariates tested
☐	☒	A description of any assumptions or corrections, such as tests of normality and adjustment for multiple comparisons
☐	☒	A full description of the statistical parameters including central tendency (e.g. means) or other basic estimates (e.g. regression coefficient) AND variation (e.g. standard deviation) or associated estimates of uncertainty (e.g. confidence intervals)
☐	☒	For null hypothesis testing, the test statistic (e.g. *F*, *t*, *r*) with confidence intervals, effect sizes, degrees of freedom and *P* value noted<br>*Give P values as exact values whenever suitable.*
☒	☐	For Bayesian analysis, information on the choice of priors and Markov chain Monte Carlo settings
☒	☐	For hierarchical and complex designs, identification of the appropriate level for tests and full reporting of outcomes
☐	☒	Estimates of effect sizes (e.g. Cohen's *d*, Pearson's *r*), indicating how they were calculated

Our web collection on statistics for biologists contains articles on many of the points above.

Software and code

Policy information about availability of computer code

Data collection	This work was conducted using published summary statistics from UK Biobank whole-exome sequencing (WES) based GWAS (Backman et al. 2021), and individual level genotypic and phenotypic data accessed through the UK Biobank application ID 81499.
Data analysis	Ancestry inference was performed using KING (v2.3.2). Principal component analysis was performed on individual level genotypic data using pcapred (v0.1.0) and flashpcaR (v2.1) in R version 4.4.0. Haplotype components computed for individuals under UKB application ID 81499 were supplied by the authors of SparsePainter and PBWTpaint (Yang et al. 2024), generated as described in the associated publication. Polygenic scores were generated using PLINK (v2). All further statistical analyses were conducted in R using publicly available analysis libraries, including: cansic (v0.9.0), sf (v1.0-20), spdep (v1.3-11), TwoSampleMR (v0.6.14), lmtest (v0.9-40) and sandwich (3.1-1).<br><br>Analysis scripts used to generate the results reported are publicly available through GitHub https://github.com/alhbiostat/rare-variant-bias.git (DOI: https://doi.org/10.5281/zenodo.19558747). Rare variant polygenic confounding simulations included in the Supplementary Notes are available at https://github.com/alhbiostat/rare-polygenic (DOI: https://doi.org/10.5281/zenodo.19558818).

For manuscripts utilizing custom algorithms or software that are central to the research but not yet described in published literature, software must be made available to editors and reviewers. We strongly encourage code deposition in a community repository (e.g. GitHub). See the Nature Portfolio guidelines for submitting code & software for further information.

Data

Policy information about availability of data

All manuscripts must include a data availability statement. This statement should provide the following information, where applicable:

- Accession codes, unique identifiers, or web links for publicly available datasets
- A description of any restrictions on data availability
- For clinical datasets or third party data, please ensure that the statement adheres to our policy

UK Biobank population exome summary statistics for complex traits are provided as supplemental data with the publication by Backman et al.26 and have been deposited within the GWAS Catalogue (https://www.ebi.ac.uk/gwas/); see Backman et al.26 Supplementary Data Table 4 for all study accession numbers (https://www.nature.com/articles/s41586-021-04103-z#Sec18). Within-sibship model summary statistics for top associated rare variants are available in Supplementary Data 1 with GWAS Catalogue accession numbers linking to full summary statistics. Individual level genetic and phenotypic data for UK Biobank participant are accessible by approved researchers; for more information on data access visit https://www.ukbiobank.ac.uk/use-our-data/. Area boundaries used for mapping UK counties and unitary authorities were taken from the December 2023 ultra-generalised UK vector boundaries from the Office for National Statistics (https://geoportal.statistics.gov.uk/datasets/countries-december-2023-boundaries-uk-buc/about). Source data for figures is provided with this paper within a source data file.

Research involving human participants, their data, or biological material

Policy information about studies with human participants or human data. See also policy information about sex, gender (identity/presentation), and sexual orientation and race, ethnicity and racism.

Reporting on sex and gender	Biological sex was controlled for by inclusion of sex as a covariate in all genetic analyses. No sex stratified analyses were performed.
Reporting on race, ethnicity, or other socially relevant groupings	Analyses were performed on individuals of genetically predicted European ancestry, determined using KING by projecting UK Biobank participant genotypes onto the 1000 Genomes Project ancestry reference set. Indices constructed from socio-demographic parameters included the 'Index of Multiple Deprivation' (UKB field ID 26410), assigned based on an individual's postcode at recruitment and area specific Townsend Deprivation Index Score, among other subdomains, as described (https://biobank.ndph.ox.ac.uk/showcase/field.cgi?id=26410), and 'Education Score', taken from the data field 'Years in Education' (UKB field ID 6138) scaled as per the International Standard Classification of Education (ISCED).
Population characteristics	The UK Biobank is a population cohort of individuals aged between 40 and 69 years and living in England, Scotland and Wales at recruitment.
Recruitment	Detailed information on the recruitment of participants into the UK Biobank is provided online at https://www.ukbiobank.ac.uk/learn-more-about-uk-biobank/about-us
Ethics oversight	Individual level data and summary statistics were taken from the UK Biobank (UKB), a UK population cohort recruited from the general population aged 40 to 69 years residing in the UK between the years of 2006 and 2010. Participants provided written informed consent for collected physiological, anthropometric, sociodemographic and biological (including genetic) data, and linked healthcare records, to be used for research purposes. Ethical approval was granted by the NHS National Research Ethics Service North West (16/NW/0274) and data was accessed under approved Application ID 81499. Full details of the UKB are provided online: https://www.ukbiobank.ac.uk/about-our-data/.

Note that full information on the approval of the study protocol must also be provided in the manuscript.

Field-specific reporting

Please select the one below that is the best fit for your research. If you are not sure, read the appropriate sections before making your selection.

☒ Life sciences ☐ Behavioural & social sciences ☐ Ecological, evolutionary & environmental sciences

For a reference copy of the document with all sections, see nature.com/documents/nr-reporting-summary-flat.pdf

Life sciences study design

All studies must disclose on these points even when the disclosure is negative.

Sample size	Analyses using individual level genetic data were performed on n=306,991 individuals of genetically determined European ancestry (including n=38,601 first degree siblings) from the UK Biobank. A subset of n=279,390 unrelated individuals of European ancestry born in the UK were used in analyses involving spatial mapping of birthplace.
Data exclusions	Individuals in the UK Biobank are predominantly of white European decent, and analyses performed in this ancestral group are best powered. Individuals of genetically determined non-European ancestry were excluded from analyses to minimise confounding of studied parameters by broad-scale ancestral genetic variation. The smaller size of non-European cohorts in the UK Biobank reduced the feasibility of conducting rare variant analyses in these groups, particularly when sub-setting to first-degree siblings. There is presently a pronounced imbalance in the representation of global ancestral diversity in genetic cohorts that needs to be rectified to ensure advances made through genetic research are applicable to and beneficial for all.

Replication	We did not seek to replicate the analyses performed here in cohorts beyond the UK Biobank as we do not currently have access to other large scale population cohorts with exome sequencing data available.
Randomization	No randomisation of study participants was performed. Correction for participant age and sex was included in all relevant statistical analyses.
Blinding	This study was based on observational data, blinding was not appropriate

Reporting for specific materials, systems and methods

We require information from authors about some types of materials, experimental systems and methods used in many studies. Here, indicate whether each material, system or method listed is relevant to your study. If you are not sure if a list item applies to your research, read the appropriate section before selecting a response.

Materials & experimental systems

n/a	Involved in the study
☒	☐ Antibodies
☒	☐ Eukaryotic cell lines
☒	☐ Palaeontology and archaeology
☒	☐ Animals and other organisms
☒	☐ Clinical data
☒	☐ Dual use research of concern
☒	☐ Plants

Methods

n/a	Involved in the study
☒	☐ ChIP-seq
☒	☐ Flow cytometry
☒	☐ MRI-based neuroimaging

Plants

Seed stocks	*Report on the source of all seed stocks or other plant material used. If applicable, state the seed stock centre and catalogue number. If plant specimens were collected from the field, describe the collection location, date and sampling procedures.*
Novel plant genotypes	*Describe the methods by which all novel plant genotypes were produced. This includes those generated by transgenic approaches, gene editing, chemical/radiation-based mutagenesis and hybridization. For transgenic lines, describe the transformation method, the number of independent lines analyzed and the generation upon which experiments were performed. For gene-edited lines, describe the editor used, the endogenous sequence targeted for editing, the targeting guide RNA sequence (if applicable) and how the editor was applied.*
Authentication	*Describe any authentication procedures for each seed stock used or novel genotype generated. Describe any experiments used to assess the effect of a mutation and, where applicable, how potential secondary effects (e.g. second site T-DNA insertions, mosiacism, off-target gene editing) were examined.*
