# Supplementary material for: Evaluating confounding in rare variant genome wide association studies
Source: Nat Commun. 2026 May 29;17:7002. doi: 10.1038/s41467-026-73776-9 (PMC13392019; doi:10.1038/s41467-026-73776-9)
Supplement: Supplementary file 6 — Transparent Peer Review file [file 41467_2026_73776_MOESM6_ESM.pdf]

nature portfolio

Peer Review File

Evaluating Confounding in Rare Variant Genome Wide Association Studies

Corresponding Author: Dr Aimee Hanson

This manuscript has been previously reviewed at another journal. This document only contains information relating to versions considered at Nature Communications.

This file contains all reviewer reports in order by version, followed by all author rebuttals in order by version.

Version 0:

Reviewer comments:

Reviewer #2

(Remarks to the Author)
The reviewers made several textual and analytical changes in response to my comments and I appreciate the good-faith engagement. We just philosophically disagree on certain points (e.g. the novelty of familial polygenic confounding as a mechanism of confounding and the comparison of rPCs and HCs in correcting for population structure), which is fine.

But their response does not address the core interpretational issue raised regarding the simulations. Setting aside the implausibility of 10,000 rare variants arising in a single individual from a family in the tails of the phenotypic distribution, the r2 between rare variants and PGS is perhaps only higher than the median common variant r2 in the first two generations (Fig. 5c). So I do not understand the statement that “The reviewer’s simulations only show associations for generation 1, when there is a single carrier of the de novo mutation. Therefore, it might not be surprising that r2 is low for the rare variant at generation 1, which is why we conducted the forward in time multi-generation simulation, in which these rare variants are randomly inherited by members of subsequent generations, alongside a shared component of the PGS, and can have expected r2 larger than the expected r2 of common causal variants carried by many members of the population.” And as I mentioned in one of my earlier reviews, what is more useful in practice is to know the false-positive rate or level of inflation expected in the effect of a rare variant given this level of correlation. I think this information would help the manuscript move past the qualitative framing of “this problem exists” – which we can agree on and I believe known – towards “here’s how big of an issue this is”.

Nevertheless, the paper is an important contribution in terms of the scale and rigor of the empirical analyses performed.

(Remarks on code availability)

Reviewer #3

(Remarks to the Author)
I previously reviewed this manuscript for a different journal, and the authors have incorporated my suggestions (and those of the other reviewers) in a way that has improved the manuscript substantially. I have two small remaining comments, both to do with the mechanism underlying the authors' "familial polygenic confounding". These would simply require some clarification in the text, not any new work.

"Familial polygenic confounding" is the idea that a rare variant might have arisen, by chance, by mutation within a lineage that has an unusually large, or unusually small, polygenic score (PGS) for the trait. The rare variant would then be associated with the trait, without necessarily having a causal effect on it.

First, I agree with Reviewer #2 that this is just a special case of genetic stratification. The authors need to do a better job of

explaining why rare-common variant genetic stratification (their case) is more likely, or more pernicious, than common-common variant genetic stratification (the case usually considered).

Second, genetic stratification arises, by definition, because of LD between variants (noncausal-causal, leading to spurious effects, or causal-causal, leading to biased effects). Yet the authors at several points in the manuscript (and in response to my previous review and that of Reviewer #2) make statements that seem to downplay the possibility of LD between the rare variant and causal/PGS variants elsewhere in the genome. Examples:
- In response to Reviewer #2: "there is no LD between this variant and the PGS variants that fall on the other 21 chromosomes"
- In response to my previous review: "there was no detectable LD between any of these three rare variants and any of the individual common variants used in the PGS (on other chromosomes)"
- lines 256-258 of the revised ms: "There was no long-range, cross-chromosomal LD detected between these variants and any of the remaining individual common variants used in PGS generation."

As I pointed out in my previous review, because the PGS is a weighted sum of genotypes across loci, any association between a rare variant and the PGS is, by definition, a reflection of LD between the rare variant and variants included in the PGS. Indeed, the covariance between the PGS and the genotype at the rare variant's locus is simply a weighted sum of LD coefficients. The authors' statements about there being no long-range LD thus have the potential to be misleading. In their response to my previous comment along these lines, the authors indicate that what they mean by such statements is that no *individual* (pairwise) LD coefficients are statistically distinguishable from zero. But I'm not sure why this is a relevant fact to report, given that it is the (weighted) sum of the LD values, not the individual values themselves, that matter, and we know this weighted sum to be nonzero.

(Remarks on code availability)

Version 1:

Reviewer comments:

Reviewer #2

(Remarks to the Author)
I have no further comments for the authors. congrats!

(Remarks on code availability)

Reviewer #3

(Remarks to the Author)
The author have adequately addressed my final (small) concerns.

(Remarks on code availability)

Open Access This Peer Review File is licensed under a Creative Commons Attribution 4.0 International License, which permits use, sharing, adaptation, distribution and reproduction in any medium or format, as long as you give appropriate credit to the original author(s) and the source, provide a link to the Creative Commons license, and indicate if changes were made.
In cases where reviewers are anonymous, credit should be given to 'Anonymous Referee' and the source.
The images or other third party material in this Peer Review File are included in the article's Creative Commons license, unless indicated otherwise in a credit line to the material. If material is not included in the article's Creative Commons license and your intended use is not permitted by statutory regulation or exceeds the permitted use, you will need to obtain permission directly from the copyright holder.
To view a copy of this license, visit https://creativecommons.org/licenses/by/4.0/

We thank both reviewers for evaluating this work a third time, and for their ongoing contribution to the strengthening of this manuscript. This has been a very valuable engagement, and we appreciate the time it has taken. We hope we have satisfactorily addressed these remaining concerns:

Reviewer #2 (Remarks to the Author):
The reviewers made several textual and analytical changes in response to my comments and I appreciate the good-faith engagement. We just philosophically disagree on certain points (e.g. the novelty of familial polygenic confounding as a mechanism of confounding and the comparison of rPCs and HCs in correcting for population structure), which is fine.

But their response does not address the core interpretational issue raised regarding the simulations. Setting aside the implausibility of 10,000 rare variants arising in a single individual from a family in the tails of the phenotypic distribution, the r2 between rare variants and PGS is perhaps only higher than the median common variant r2 in the first two generations (Fig. 5c). So I do not understand the statement that “The reviewer’s simulations only show associations for generation 1, when there is a single carrier of the de novo mutation. Therefore, it might not be surprising that r2 is low for the rare variant at generation 1, which is why we conducted the forward in time multi-generation simulation, in which these rare variants are randomly inherited by members of subsequent generations, alongside a shared component of the PGS, and can have expected r2 larger than the expected r2 of common causal variants carried by many members of the population.” And as I mentioned in one of my earlier reviews, what is more useful in practice is to know the false-positive rate or level of inflation expected in the effect of a rare variant given this level of correlation. I think this information would help the manuscript move past the qualitative framing of “this problem exists” – which we can agree on and I believe known – towards “here’s how big of an issue this is”.

Nevertheless, the paper is an important contribution in terms of the scale and rigor of the empirical analyses performed.

We appreciate that there remains some disagreement about the conduct of the simulations, and we have tried to address these, justifying our reasoning for the parameters used in previous responses. We do acknowledge that these simulations best stand to illustrate the mechanism of familial confounding, rather than support definitive claims as to how exactly this may manifest in real world population studies. The reviewer has been right to emphasise the ways in which these simulations may deviate from the use of realistic real-world parameters. We also acknowledge that we do not model factors, such as sample size and environmental effects, which will impact observed rare variant-trait correlations in a population. We do however believe these simulations help highlight the far greater liability of null rare variants to biasing by this mechanism than common variants, and demonstrate how population relatedness structure, and mechanisms inducing phenotypic correlation between even distant family members, play into this. As individuals in the phenotypic extremes carrying linage-specific mutations will exist in any population genetic study, and at worst acute confounding could be expected to yield highly significant false positive associations, we wish readers and analysts to be on alert for this as the field moves towards WES and WGS based population genetic studies.

Given the concerns raised by the reviewer, we have decided to move the simulations and the associated figures to the supplement. We still refer to them in the text, but we now address their limitations, and emphasise that the extent to which familial confounding is problematic will be dependent on the degree of relatedness in a study sample and how strongly correlated the phenotypic value of distant relatives remains through e.g. assortative mating or shared environmental effects, all of which will be population and study specific. We understand that this is not a quantitative statement as the reviewer requests, but given the points raised above, this is not straightforward to provide. We have tempered our wording throughout to remove suggestions that this mechanism is entirely novel,

and have shifted our framing towards emphasising why it is more problematic in rare than common variant GWAS.

Location	Original text (new text)
**Abstract**<br>Line 21, Page 1 (in tracked changes doc)	We explore the risk of elevated false discovery rates for recent variants private to extended families sharing polygenic liability to extreme phenotypes, as well as through local linkage with common causal variants. ~~Through simulations, we show that recent private variants are susceptible to elevated false discovery rates due to familial confounding and linkage with common causal variants, which may drive some reported rare variant height associations.~~ Overall, we consider the complex confounding mechanisms that can impact rare variant studies and demonstrate family-based approaches enabling critical sensitivity analyses.
**Results**<br>Line 229, Page 7	The latter, which we refer to here as ~~have termed~~ familial polygenic confounding, can be considered an extreme version of confounding by cryptic relatedness (CR)[30], and may occur in situations where *de novo* variants, private to a family lineage, emerge within a genomic landscape containing a high common variant burden for a polygenic trait, underlying an extreme phenotype.
**Results**<br>Line 267, Page 8	Supplementary Note 2 details simulations we conducted to investigate the liability to familial polygenic confounding of *de novo* variants transmitted through generations. Supplementary Fig.12 and 13 show that simulated *de novo* null rare variants emerging in a lineage with an extreme mean phenotypic value can show substantial correlations with a measured trait or trait PGS (Supplementary Fig.13) ~~even exceeding that of causal common variants~~ when trait heritability and assortative mating are high, though other unmodelled factors, including sample size and environmental factors, will likely influence the strength of this correlation.
**Results**<br>Line 281, Page 9	Thus, our findings demonstrate that false positive rare variant associations can arise due to local LD with common causal variants, and our simulations suggest that familial polygenic confounding may result in spurious associations with rare variants private to extended families with extreme phenotypes. In practice, the consequence of the latter mechanism will depend on the degree of relatedness in the study population, and the extent to which phenotypic correlations between distal relatives are preserved by assortative mating or shared environmental factors. We detect evidence of potentially spurious height-associated rare variants which show strong associations with the common variant height PGS independent of local LD, and promote sensitivity analyses assessing rare variant-PGS associations to flag potentially unreliable rare variant effects confounded by relatedness.
**Discussion**<br>Line 376, Page 11	Beyond genetic confounding, which also strongly impacts common variant GWAS, we ~~present a basis for an independent~~ explore the mechanism of familial polygenic confounding, a biasing phenomenon expected to be unique to rare variant studies entirely due to the familial restriction of very rare variants. Conceptually, this issue is akin to, but more acute than the known issue of cryptic relatedness, though it has been argued that the shared ancestry and environment of related individuals in common variant GWAS is unlikely to induce substantial genotype-phenotype correlation in reality[30]. In GWAS of rare variants, it is possible for a single lineage to carry the entirety of a genetic signal, and every variant private to an extended family at the extreme tails of the PGS distribution would be susceptible to strong confounding due to what is essentially an issue of sharp population structure.
**Discussion**<br>Line 403, Page 12	Though further empirical evidence is required to assess the precise scenarios in which such confounding may arise, it is important to consider as a driver of false positives as the rapid growth of sequenced population cohorts facilitates further rare variant association studies.
	Further, we ~~present~~ consider acute familial polygenic confounding as a mechanism with the potential to induce strongly confounded single rare variant association with private variants in outlying families, which will likely require more effective correction for granular relatedness structure to address.

We have also regenerated the results shown in Supplementary Fig. 13 using 100 private variants, which better approximates the de novo mutation rate in humans (1.1-1.3x10^{-8} substitutions per nucleotide per generation, PMID: 22914163 and 40089484).

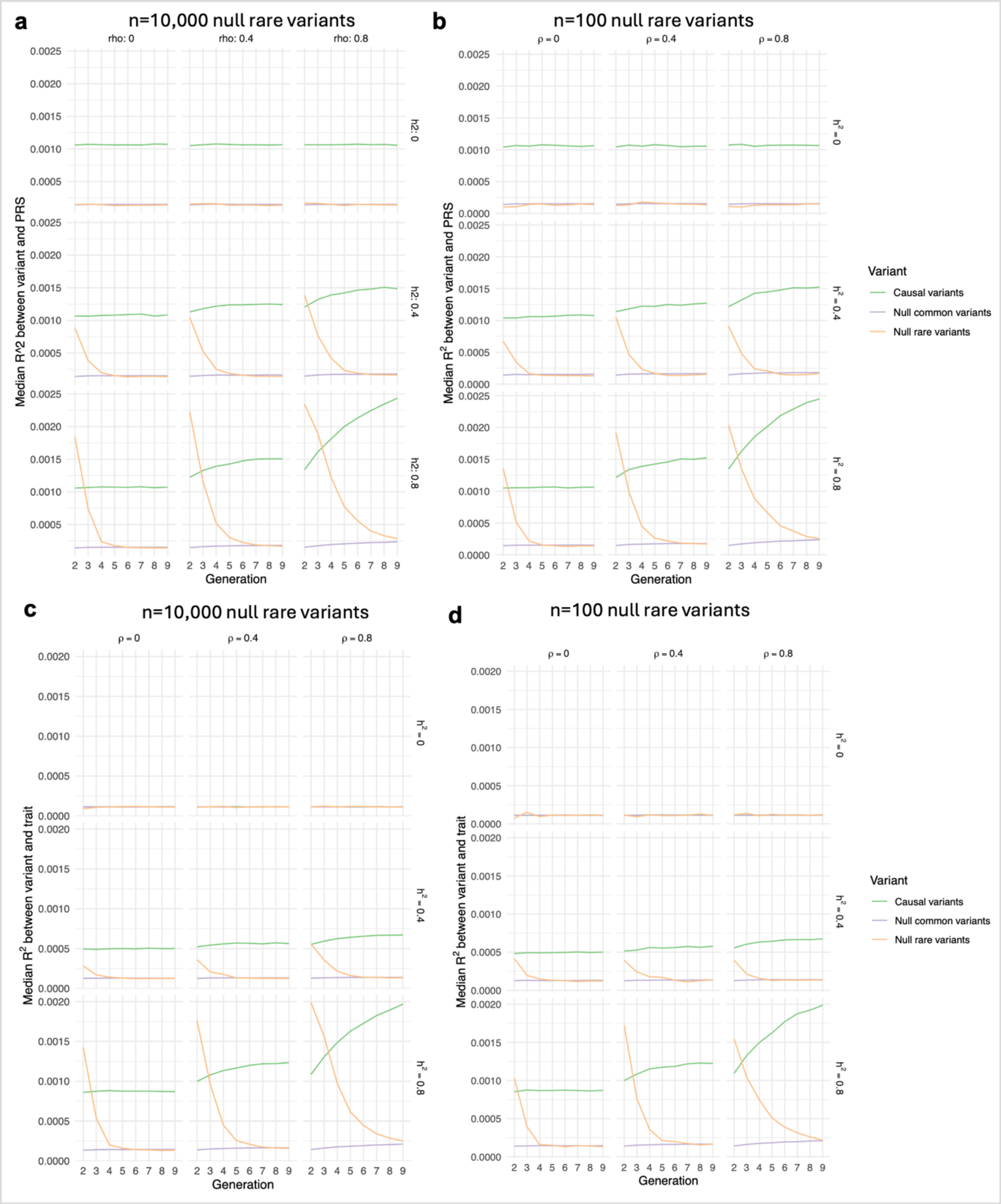

Reviewer #3 (Remarks to the Author):

I previously reviewed this manuscript for a different journal, and the authors have incorporated my suggestions (and those of the other reviewers) in a way that has improved the manuscript substantially. I have two small remaining comments, both to do with the mechanism underlying the authors' "familial polygenic confounding". These would simply require some clarification in the text, not any new work.

"Familial polygenic confounding" is the idea that a rare variant might have arisen, by chance, by mutation within a lineage that has an unusually large, or unusually small, polygenic score (PGS) for the trait. The rare variant would then be associated with the trait, without necessarily having a causal effect on it.

First, I agree with Reviewer #2 that this is just a special case of genetic stratification. The authors need to do a better job of explaining why rare-common variant genetic stratification (their case) is more likely, or more pernicious, than common-common variant genetic stratification (the case usually considered).

We hope we have now emphasised this sufficiently with some modifications to the discussion, including in response to reviewer 2 above:

Location	Original text (new text)
**Discussion**<br>Line 369, Page 11 (in tracked changes doc)	Beyond genetic confounding, which also strongly impacts common variant GWAS, we explore the mechanism of familial polygenic confounding, a biasing phenomenon expected to be unique to rare variant studies entirely due to the familial restriction of very rare variants. Conceptually, this issue is akin to, but more acute than the known issue of cryptic relatedness, though it has been argued that the shared ancestry and environment of related individuals in common variant GWAS is unlikely to induce substantial genotype-phenotype correlation in reality[30]. In GWAS of rare variants, it is possible for a single lineage to carry the entirety of a genetic signal, and every variant private to an extended family at the extreme tails of the PGS distribution would be susceptible to strong confounding due to what is essentially an issue of sharp population structure. Such confounding would also be expected in situations where an extreme phenotype arises through a shared environment (the most acute example of the spatial confounding explored above), though environmental effects would feasibly be less specifically restricted to dispersed distant relatives than is the inheritance of a shared polygenic background. Through simulations, we show that when null rare variants emerge within a family with a high polygenic burden, underlying an extreme phenotype, they correlate strongly with polygenic trait liability and trait expression, which takes multiple generations to decay when heritability and assortative mating are high. This correlation is greater than that expected between a causal rare variant and the polygenic component of a trait due to assortative mating, such that identifying strong rare variant associations with the PGS (with the target chromosome excluded) presents a practical means to distinguish unreliable results. Common null variants are unlikely to be biased by this mechanism due to being widely distributed across a population of diverse genetic background. Population stratification could theoretically also induce rare variant-PGS correlations, which these sensitivity analyses would also detect.

Second, genetic stratification arises, by definition, because of LD between variants (noncausal-causal, leading to spurious effects, or causal-causal, leading to biased effects). Yet the authors at several points in the manuscript (and in response to my previous review and that of Reviewer #2) make statements that seem to downplay the possibility of LD between the rare variant and causal/PGS variants elsewhere in the genome. Examples:
- In response to Reviewer #2: "there is no LD between this variant and the PGS variants that fall on the other 21 chromosomes"
- In response to my previous review: "there was no detectable LD between any of these three rare variants and any of the individual common variants used in the PGS (on other chromosomes)"
- lines 256-258 of the revised ms: "There was no long-range, cross-chromosomal LD detected between these variants and any of the remaining individual common variants used in PGS generation."

As I pointed out in my previous review, because the PGS is a weighted sum of genotypes across loci, any association between a rare variant and the PGS is, by definition, a reflection of LD between the rare variant and variants included in the PGS. Indeed, the covariance between the PGS and the genotype at the rare variant's locus is simply a weighted sum of LD coefficients. The authors' statements about there being no long-range LD thus have the potential to be misleading. In their response to my previous comment along these lines, the authors indicate that what they mean by such statements is that no *individual* (pairwise) LD coefficients are statistically distinguishable from zero. But I'm not sure why this is a relevant fact to report, given that it is the (weighted) sum of the LD values, not the individual values themselves, that matter, and we know this weighted sum to be nonzero.

We think we understand the distinction the reviewer is trying to make here. The leave-one-chromosome-out analysis is based on the assumption that there should be no LD on the population level between rare variants and common PRS variants on differing chromosomes, and if associations with the loo-PRS arise this could flag spurious associations. One mechanism by which long-range LD could arise between causal variants is through assortative mating, though we show through simulations in Supplementary Fig. 15 this is likely to be negligible. Long-range LD could possibly also arise in complex situations of gene-gene epistasis, which we endeavoured to test by assessing LD between variants in a pairwise fashion. The reviewer is correct however in that we do not make this logic clear, and LD metrics, including both R^2 and D-prime, are unreliable to interpret between variants with large frequency differences and very rare variants. We understand that the reviewer is saying that observing an association between a rare variant and the PRS is a reflection of LD, though, in the context of polygenic confounding, this LD is driven by a family lineage. We have removed the sentence "There was no long-range, cross-chromosomal LD detected between these variants and any single common variants used in PGS generation" as we agree this does not add useful insight.
